# High-Speed Tracer Analysis of Metabolism (HS-TrAM)

**DOI:** 10.12688/wellcomeopenres.13387.2

**Published:** 2018-08-22

**Authors:** Thomas Brendan Smith, Kamlesh Patel, Haydn Munford, Andrew Peet, Daniel A. Tennant, Mark Jeeves, Christian Ludwig

**Affiliations:** 1 Institute of Metabolism and Systems Research, University of Birmingham, West Midlands, UK; 2 Institute of Cancer and Genomic Sciences, University of Birmingham, West Midlands, UK; 3 Birmingham Children’s Hospital NHS Foundation Trust, West Midlands, UK

**Keywords:** NMR, stable-isotope tracing, 13C, 15N, splitting enhancement, metabolism, tracer

## Abstract

Tracing the fate of stable isotopically-enriched nutrients is a sophisticated method of describing and quantifying the activity of metabolic pathways. Nuclear Magnetic Resonance (NMR) spectroscopy offers high resolution data in terms of resolving metabolic pathway utilisation. Despite this, NMR spectroscopy is under-utilised due to length of time required to collect the data, quantification requiring multiple samples and complicated analysis. Here we present two techniques, quantitative spectral filters and enhancement of the splitting of ^13^C signals due to homonuclear ^13^C, ^13^C or heteronuclear ^13^C, ^15^N J-coupling in ^1^H, ^13^C-HSQC NMR spectra. Together, these allow the rapid collection of NMR spectroscopy data in a quantitative manner on a single sample. The reduced duration of HSQC spectra data acquisition opens up the possibility of real-time tracing of metabolism including the study of metabolic pathways *in vivo*. We show how these techniques can be used to trace the fate of labelled nutrients in a whole organ model of kidney preservation prior to transplantation using a porcine kidney as a model organ. In addition, we show how the use of multiple nutrients, differentially labelled with ^13^C and ^15^N, can be used to provide additional information with which to profile metabolic pathways.

## Introduction

Investigations of metabolism in health and disease increasingly rely on tracing the use of stable isotope-enriched nutrients through the cell’s metabolic pathways. The most widely utilised technology platform to analyse the resulting complex patterns of labelling in multiple cellular metabolites is mass spectrometry (MS), due to its high sensitivity, short run times and a resulting low-cost operation ^1– 12^. Conversely, NMR spectroscopy is relatively under-utilised, despite being able to provide higher resolution information on the conversion of synthetically produced stable isotopes of nutrients are incorporated into cellular metabolites ^12– 19^. This is because NMR spectroscopy has historically suffered from low sensitivity, long acquisition times and the need for complex analytical tools.

NMR spectroscopy is, however, ideally suited to answering some of the more pressing questions about metabolic control in health and disease. We currently have limited knowledge about the compartmentalisation of metabolic pathways in metabolically-active organelles, such as mitochondria, and therefore whether the same metabolite is selectively utilised for distinct purposes in different compartments ^20^. Given the recent drive to target metabolism in various diseases, understanding the control of metabolism by different tissues is critical to the ability to select specific therapies which target the desired pathways within appropriate cellular compartments. While sample analysis by high-resolution NMR spectroscopy is performed *ex vivo* or *in vitro*, the data obtained provide information on metabolic pathways *in vivo*.

**Figure 1.**
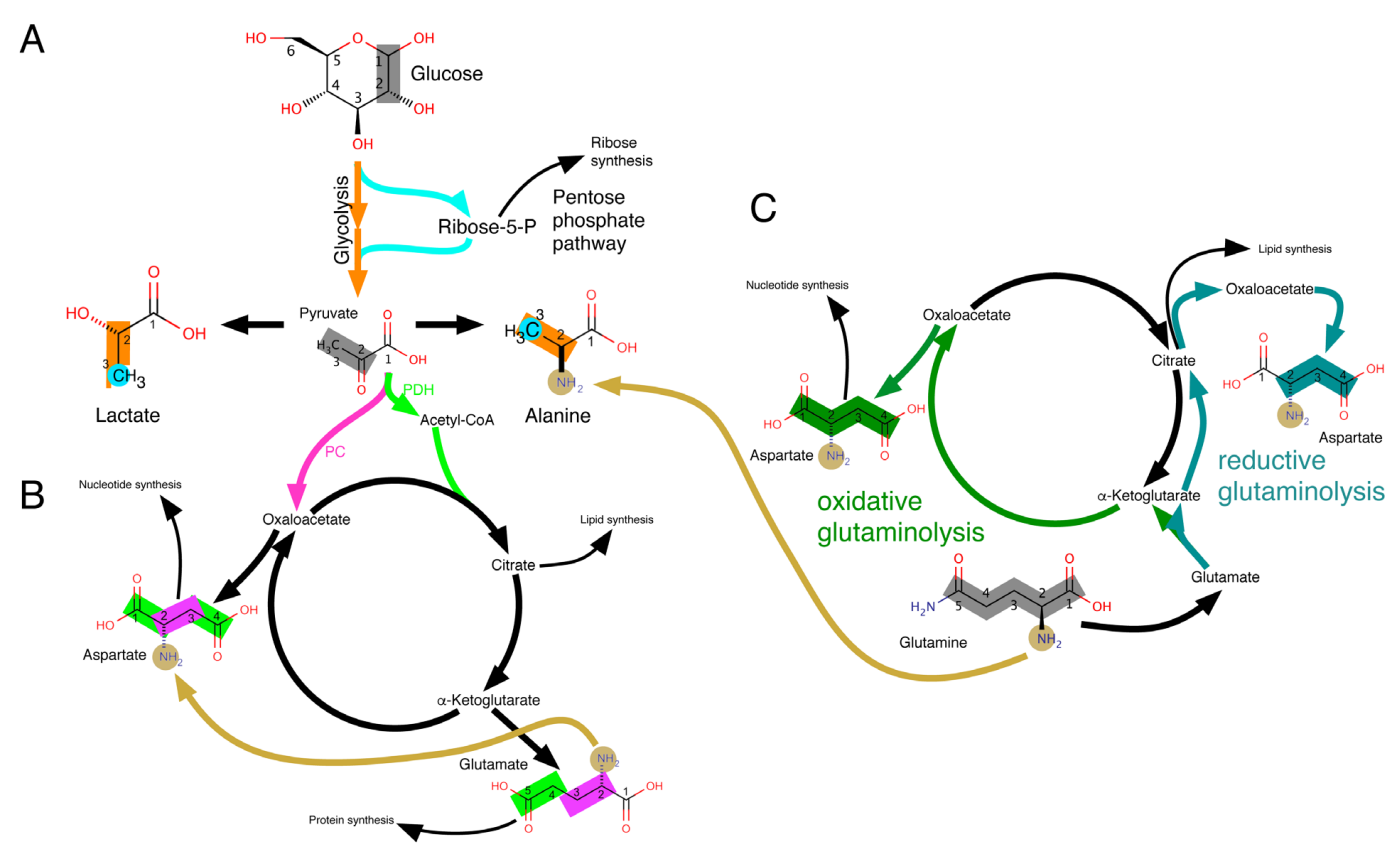
Tracing of metabolic pathways. The labelling patterns arising from [1,2- ^13^C] glucose ( **A** & **B**) as well as from [U- ^13^C, ^15^N] glutamine ( **C** for ^13^C labelling and **A**– **C** for ^15^N labelling) are shown. Metabolism of [1,2- ^13^C] glucose leads to distinctive labelling patterns in lactate and alanine ([2,3- ^13^C] lactate/alanine when using glycolysis and [3- ^13^C] lactate/alanine when using the pentose phosphate shunt, PPP) (Panel **A**). Similarly, glutamate and aspartate express different labelling patterns from [1,2- ^13^C] glucose, depending whether they were synthesised via pyruvate dehydrogenase (PDH; resulting in [4,5- ^13^C] glutamate) or via the pyruvate carboxylase (PC; resulting in [2,3- ^13^C] aspartate) route (Panel **B**). Metabolisation of labelled glutamine can reveal other anaplerotic pathway activities such as reductive carboxylation (Panel **C**).

Stable isotope-enriched metabolic precursors, such as glucose or glutamine, are employed as metabolic tracers. These synthetically produced nutrients are enriched in isotopes with a low natural abundance, such as ^13^C or ^15^N. Despite the fact that metabolites can arise from multiple sources, the contribution of the different metabolic pathways to the synthesis of this metabolite can be determined through the analysis of the ^13^C and/or ^15^N distribution within the metabolite ( Figure 1). The couplings are visualised in the indirect dimension of an HSQC spectrum allowing the determination of the percentage incorporation of isotopic label into adjacent nuclei. While MS data does not need a reference sample to distinguish between labelled and unlabelled metabolites, it is not always possible to derive the exact distribution of labelled nuclei within a molecule. In contrast, NMR spectroscopy data can resolve label distribution at the atomic level, enabling a clearer picture of the label distribution in metabolites.

Our recently published combined NMR spectroscopy and MS approach (CANMS) harnesses the strengths of both modalities to produce highly-resolved metabolism information in the form of metabolite isotopomers ^19^. The detailed interpretation of MS isotopologue data, when using MS data in isolation, requires use of a pre-defined metabolic model. In contrast, the integrated analysis of NMR spectroscopy and MS data makes fewer assumptions about the metabolic network, providing a more accurate insight into relative pathway contributions than is possible with current established methods or the independent analysis of MS or NMR spectroscopy data alone. For example, proton-less carbon atoms do not give rise to a signal in 2D-HSQC NMR spectra, although incomplete information on those carbons is available via splitting of adjacent carbon nucleus signals. The combination of NMR spectroscopy and MS analysis fills this gap as the MS data provides information on the amount of single carbon labelling into those carbon nuclei via “m+x” isotopologues. [1,2- ^13^C] glucose is the optimal tracer to assess metabolic flux through glycolysis vs pentose phosphate pathway (PPP) shunting back into glycolysis. While the glycolytically derived isotopomer of lactate is [2,3- ^13^C] lactate, the PPP derived isotopomers can be [3- ^13^C], [1- ^13^C] or [1,3- ^13^C] lactate. Although the first isotopomer can be assessed with NMR spectroscopy data, the other two isotopomers include labelling in C(1), which HSQC NMR spectroscopy is “blind” to. In these cases, MS data adds new information to the NMR spectroscopy data by contributing the isotopologues NMR spectroscopy is not able to detect, while NMR spectroscopy adds to the MS data by differentiating between [1,3- ^13^C] and [2,3- ^13^C] lactate.

A major drawback of utilising ^13^C- ^13^C scalar coupling information to derive isotopomer distributions is the time required to acquire spectra. For example, around four hours are required for the acquisition of a 2D-HSQC NMR spectrum with high-resolution in the ^13^C dimension, even when using fast, state-of-the-art non-uniform sampling (NUS) techniques.

Here we describe two developments, quantitative spectral filters and signal splitting enhancement, to facilitate and speed-up the acquisition of NMR spectra for tracer-based analysis of metabolism. Such techniques permit high throughput metabolic pathway profiling, increasing access, affordability and sensitivity when using NMR spectroscopy as an investigative modality. Additionally, these developments facilitate fast detection of ^15^N labelling, especially when combined with ^13^C tracing, thus providing extra information allowing more accurate metabolic pathway profiling.

## Methods and results

### Quantitative spectral filters for ^13^C tracer observation: 1D Spectral filters

***Experimental setup.*** A porcine kidney was procured from a slaughterhouse (FA Gill, Wolverhampton) following approximately 14 minutes warm ischaemic time (WIT) as per previous experimental methodology ^18^. No animal was killed solely for experimental purposes; all were due for human consumption, therefore no ethical approval was required. After 2 hours cold ischaemic time, kidneys were subject to 18 hours of hypothermic machine perfusion. The perfusate sample was collected after 6 hrs of perfusion and prepared for NMR analysis.

1D NMR spectra were acquired using a Bruker Avance III 600 MHz NMR spectrometer equipped with a 5mm z-PFG TCI Cryoprobe. 128 transients were acquired for each spectrum with a 5 s interscan relaxation delay. A total of 32768 data points with a spectral width of 12 ppm was acquired for each FID using an adiabatic bi-level decoupling scheme to suppress ^1^H, ^13^C J-coupling during acquisition ^21^. While decoupling for this long (2.25 s) was possible because of the cryogenic probe and may potentially work with a room temperature probe, care must be taken as there will be significant sample heating. The sample heating can be significantly reduced, with only very minor reduction in resolution, by acquiring for only 1.125 s. In order to estimate whether a specific spectrometer with a cryoprobe can tolerate the power dissipation originating from the decoupling sequence specific attention should be paid to the cryogas heater current, which should never fall below its system specific lower limit.

The spectra were processed within the MetaboLab software package (version 2018.07182055) ^22^. A 0.5 Hz line broadening was applied with zero-filling of the data up to 131072 real data points prior to Fourier transformation. The resulting spectra were referenced using DSS and manually phase corrected. Subsequently the spectral baseline was corrected using MetaboLab’s spline baseline correction before the spectra were exported to Bruker format for metabolites to be quantified in the Chenomx software package (version 8.2, http://www.chenomx.com).

***NMR methodology.*** Despite their relative simplicity and limited resolution, 1D-NMR spectra are highly sensitive tools with which to identify and quantify metabolites. Spectral filters enable the acquisition of spectra which filter out certain signals, thereby reducing ambiguity in 1D spectra associated with attributing peaks to nuclei within metabolites. For example, one can acquire 1D ^1^H NMR spectra of protons bound to ^13^C only, simplifying signal assignment and analysis of the acquired spectra. The simplest approach to collect such spectra would be to acquire the first increment of a 2D- ^1^H, ^13^C-HSQC spectrum. However, signal intensities are not directly comparable with those in standard 1D- ^1^H NMR spectra. It is therefore not possible to directly derive ^13^C percentages based on a comparison of those spectra with standard proton 1D spectra unless only a small subset of molecules is labelled with ^13^C and one accompanying spectrum is scaled so that the majority of signals within the two spectra are of same intensity ^23^. Spectral filters such as BIRD, TANGO and POCE ^24– 28^ originated in protein NMR spectroscopy to filter out certain parts of the magnetisation and therefore quantitative data cannot be gained from resultant output spectra. Quantitative comparisons between unfiltered and filtered spectra are usually unnecessary, except for tracer-based analysis. Here we present a novel spectral filter which enables quantitative analysis of resultant spectra from single samples, enriched with ^13^C tracer.

**Figure 2.**
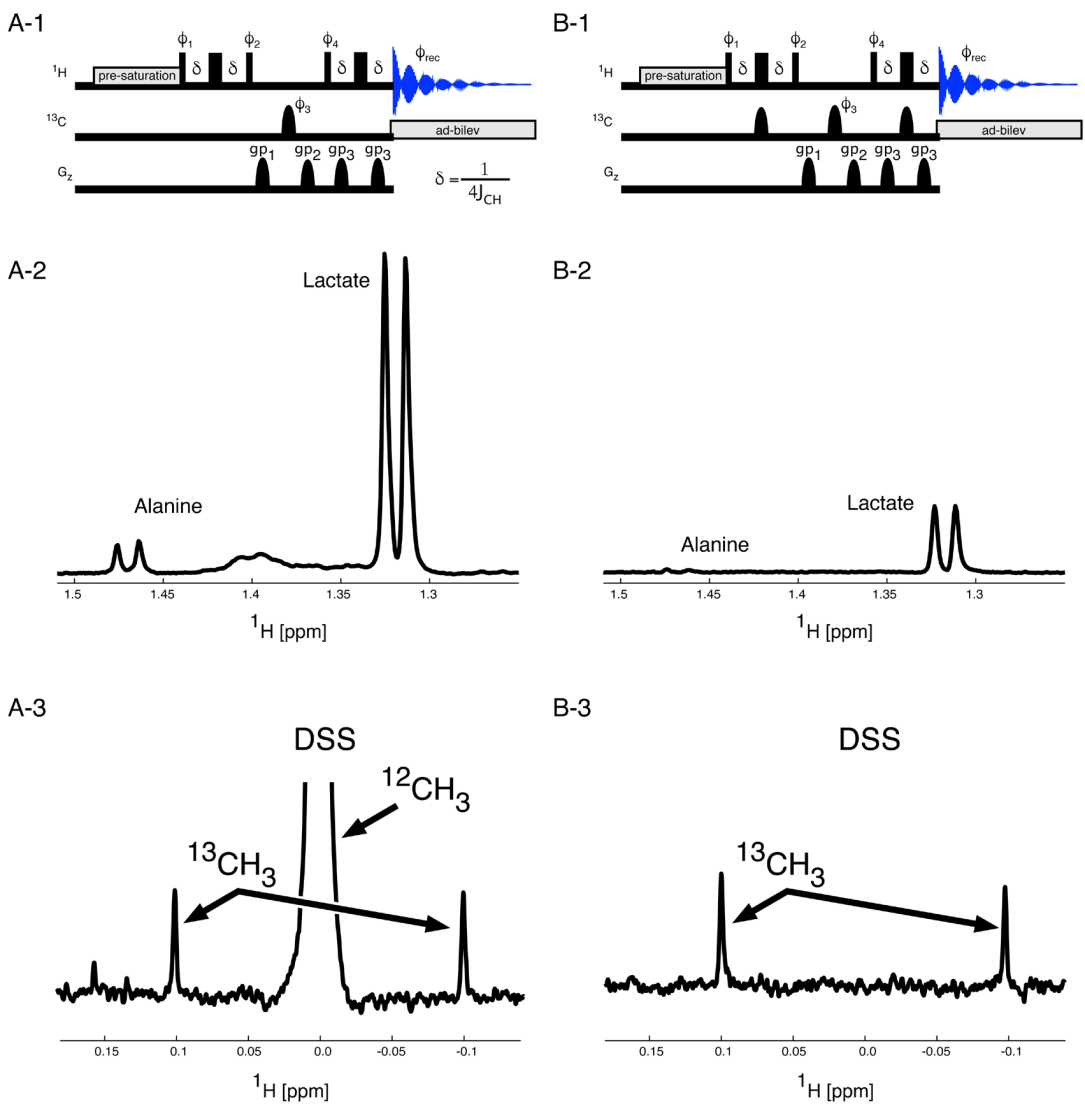
Spectral Filters in 1D spectroscopy. To determine percentage ^13^C incorporation two spectra are acquired per sample. One spectrum ( **A**) contains ^1^H signals originating from all protons (all ^1^H spectrum), while the second spectrum ( **B**) only contains signals from protons attached to a ^13^C nucleus. The central ^13^C π–pulse with phase ϕ _3_ is executed every second transient in both experiments. The proton magnetisation in the all ^1^H pulse sequence is the same for ^12^C and for ^13^C bound protons and as a consequence, all ^1^H magnetisation is longitudinal during the interval between ^1^H pulses with the phases ϕ _2_ and ϕ _4_. Because of the additional ^13^C π–pulses in the ^13^C bound ^1^H pulse sequence, the magnetisation for the two different kinds of protons develops differently. Here only the ^13^C bound ^1^H magnetisation is longitudinal in the interval between the ^1^H pulses with the phases ϕ _2_ and ϕ _4_. Therefore, the ^12^C bound ^1^H magnetisation can be destroyed using the two pulse field gradients labelled gp _1_ and gp _2_. A recovery delay of 200 µs was used after each gradient. The central ^13^C π–pulse with phase ϕ _4_ improves magnetisation selection as it is accompanied by phase changes of the ^1^H pulses and the receiver. All ^13^C π-pulses are adiabatic Chirp pulses with γB _1max_ = 10 kHz. ^1^H, ^13^C J-coupling is suppressed during acquisition using adiabatic bilevel decoupling (ad-bilev) ^21^. The pulse phases are: ϕ _1_ = x, x, -x, -x; ϕ _2_ = x, x, -x, -x for the all ^1^H spectrum ( **A-1**) and y, y, y, y for the ^13^C bound ^1^H spectrum ( **B-1**), ϕ _3_ = x, y, -x, -y, ϕ _4_ = x, -x, -x, x, y, -y, -y, y for the all ^1^H spectrum ( **A-1**) and y, -y, -y, y, -x, x, x, -x for the ^13^C bound ^1^H spectrum ( **B-1**). Panels **A-2** and **B-2** show the peaks from Alanine and Lactate methyl protons in the all ^1^H spectrum ( **A-2**) and the ^13^C bound ^1^H spectrum ( **B-2**). The scaling of the two spectra is identical allowing easy determination of the percentage incorporation of ^13^C metabolites. Panels **A-3** and **B-3** demonstrate the complete removal of the ^12^C bound proton signal from the ^13^C edited spectrum (B-3) leaving only the natural abundance ^13^C signals to be observed.

Panels A-1 and B-1 in Figure 2 show the pulse sequences implementing the quantitative spectral filter. While the central ^13^C π-pulse (phase ϕ _3_) is used only in odd numbered transients and replaced with a delay of the same length during even numbered transients, the two other ^13^C π-pulses are only used in the ^12^C filtering experiment (B-1), where ^1^H magnetisation to ^12^C neighbours is filtered out, so that only ^13^C bound ^1^H magnetisation contributes to the signal intensities in the 1D spectrum. The phase cycle ϕ _1_ changes as well between the 2 experiments, as indicated in the figure legend.

Panels A-2 and B-2 in Figure 2 depict two sample spectra from a perfusate sample where a cadaveric porcine kidney was perfused with modified University of Wisconsin machine perfusion solution (UW MPS) during a period of hypothermic machine perfusion. The standard unlabelled glucose constituent (10 mM) within classical UW MPS was replaced with 10 mM universally labelled glucose, for use as a metabolic tracer during the 18 hour perfusion.

While filters such as BIRD utilise relaxation to minimise the unwanted part of the magnetisation, methods such as TANGO or POCE generate magnetisation where ^12^C bound ^1^H atoms possess either the same or the opposite phase compared to the magnetisation of ^13^C bound ^1^H atoms, generating two different spectra. By subtraction of these two spectra, the magnetisation of ^13^C bound ^1^H atoms can then be calculated. In case of low ^13^C incorporation, as with any difference technique, subtracting two very large signals in presence of a small signal can lead to substantial artefacts. The quantitative spectral filter works slightly differently compared to TANGO and POCE as the pulse sequence depicted in Figure 2, panel B-1 makes use of two gradient pulses (gp _1_ and gp _2_) to destroy unwanted magnetisation. As an example, panels A-3 and B3 in Figure 2 show a variant of the pulse sequences where the adiabatic bilevel decoupling (ad-bilev) ^21^ has been omitted, so that the ^12^C and the ^13^C bound ^1^H signals appear separated in the spectrum. The ^12^CH _3_ signal in A-3 has been truncated to be able to visualise the ^13^C satellites, which appear with 0.5% of the peak height of the ^12^CH _3_ signal. As can be seen in panel B-3, the ^12^CH _3_ signal is completely suppressed without leaving an artefact, so that even signals from naturally occurring ^13^C alone are easily detectable in a ^13^C decoupled spectrum.

The quantitative spectral filter is invariant with respect to differential ^1^H relaxation rates or signal multiplicities. As with any J-coupling based filtering approach, the ^12^C filtered spectrum will be scaled with a factor that is proportional to sin(2πδJ _CH_) ^2^, where δ is the delay during the first and last spin echo. δ is usually set to ¼J _CH_ with J _CH_ = 145 Hz. Assuming a minimum J _CH_ of 120 Hz and a maximum J _CH_ of 165 Hz, results in a maximum downscaling of 7.2%.

### J-Coupling based splitting enhancement in 2D-NMR spectra

***Experimental setup.*** Slaughterhouse porcine kidneys (WIT-15 minutes) were cannulated and flushed with chilled Soltran solution (Baxter) as performed in clinical practice. Kidneys were placed in static cold storage en route to our laboratory, where they were immediately perfused with modified KPS-1 using the Lifeport Kidney Transporter 1.0 (Organ Recovery Systems), which has been modified to include a paediatric oxygenator. Oxygen was supplied at a flow rate of 0.7 L/min for the duration of the 24 hours perfusion period.

At the end of the perfusion period, the kidney was removed from the perfusion circuit and laterally bisected. Sections of cortex and medulla were isolated and snap frozen in liquid nitrogen. These tissues were powdered, also under liquid nitrogen, and 0.5 g was placed in 7 ml homogenisation tube (Precellys, CK28), containing 5.1 ml of HPLC grade methanol (−80°C) to quench metabolism. These were homogenised using the Precellys 24 dual homogeniser (8x 5000 rpm for 15 s). The samples were mixed with 4.65 ml deionised water and 5.1 ml HPLC grade chloroform and vigorously agitated. Biphasic separation of polar and non-polar solvents was performed by centrifugation (1300 g, 15 minutes, 4°C), after which 4.5 ml of the polar layer was aspirated and dried overnight at 35°C.

The dried extracts were resuspended in 60 µl NMR buffer (0.1 M phosphate buffer, 0.5 mM 4,4-dimethyl-4-silapentane-1-sulfonic acid, 2 mM imidazole and 10% D _2_O). These suspensions were sonicated to dissolve micro particles and then 35 µl of this solution was added to 1.7 mm NMR tubes.

^1^H, ^13^C-HSQC spectra were acquired using a Bruker Avance III 600 MHz NMR spectrometer equipped with a 1.7 mm z-PFG TCI Cryoprobe. The HSQC spectra were acquired using 2 transients per increment with echo/anti-echo gradient coherence selection and an additional pre-saturation for suppressing the water resonance during the 1.5 s interscan relaxation delay. The ^1^H dimension was acquired with a spectral width of 13 ppm using 512 complex data points. The ^13^C dimension was acquired with a spectral width of 160 ppm using 25% (2048) of 8192 data points using a non-uniform sampling scheme. The non-uniformly sampled spectra were reconstructed via the compressed sensing algorithm within the MDDNMR (version 2.5) ^29^ and processed using NMRPipe (version 9.2) ^30^. All spectra were processed without baseline correction to avoid complications in the multiplet analysis procedure.

**Figure 3.**
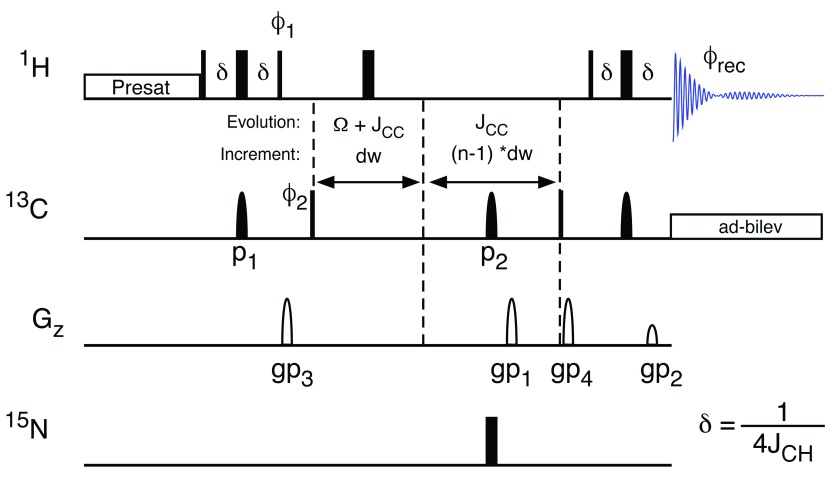
Splitting Enhanced HSQC Spectroscopy. The splitting enhancement due to J-coupling is achieved using an additional spin echo subsequent to the ^13^C evolution period. The delays in the spin-echo for the J-coupling enhancement are multiples of the dwell time (dw). While the chemical shift evolves with dw, which is determined by setting the spectral width of the spectrum, splittings are enhanced depending on the increment of the ^13^C gradient selection spin-echo. The HSQC spectrum is acquired using echo/anti-echo for quadrature detection to allow for efficient removal of artefacts in only two scans per increment. Optionally, the ^13^C, ^15^N-couplings can be scaled by the introduction of the ^15^N π-pulse simultaneously with the ^13^C π-pulse (labelled p2). ^1^H, ^13^C J-coupling is suppressed during acquisition using adiabatic bilevel decoupling (ad-bilev) ^21^. The pulse phases are: ϕ _1_ = y; ϕ _2_ = x, -x; ϕ _rec_ = x, -x.

**Figure 4.**
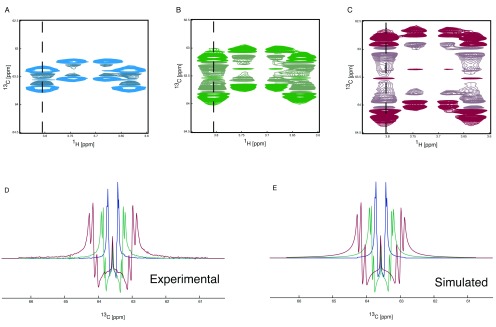
J-coupling splitting enhancement HSQC spectroscopy. ^1^H, ^13^C HSQC spectra showing the C(6) of glucose are shown ( **A**, **B** and **C**). The spectrum with no J-coupling splitting enhancement is shown in blue ( **A**), with an enhancement of two in green ( **B**) and with an enhancement of four in red ( **C**). The ^13^C trace of the HSQC spectra ( **D**), taken from the ^1^H frequency as depicted by the dashed line, clearly shows the increase in observed splitting. The J-coupling splitting enhancement is achieved using an additional spin echo subsequent to the ^13^C evolution period. The delays to achieve the scaling of the splittings are multiples of dw such the use of a delay of 3*dw will result in a J-coupling splitting enhancement of 4 (one from the t _1_ evolution and three from the J-coupling splitting enhancement spin echo). The observed splitting can be simulated ( **E**) giving the following incorporation percentages. From the no J-coupling splitting enhancement spectrum 6.8% / 41.2% / 52 % for [6- ^13^C] / [5,6- ^13^C] / [U- ^13^C], from the two-fold J-coupling splitting enhancement 6.3% / 41.4% / 52.3 % for [6- ^13^C] / [5,6- ^13^C] / [U- ^13^C] and from the four-fold J-coupling splitting enhancement 5.9% / 41.6% / 52.5 % for [6- ^13^C] / [5,6- ^13^C] / [U- ^13^C].

***NMR methodology.*** The relatively long acquisition times of 2D-HSQC spectra are necessary to generate the spectral resolution required to resolve complex multiplet patterns ^19^. Here we present a technique to manipulate the appearance of NMR multiplets in the indirect dimension of 2D-HSQC spectra. The ability to expand the splitting caused by J-coupling has previously been reported ^31, 32^. Here we apply this technique in order to negate the requirement for the collection of large number of increments in the ^13^C dimension, which, together with methods such as non-uniform sampling ^29, 33^ and variation of the repetition time ^34^ significantly reduces the time required to acquire 2D-HSQC spectra with sufficient resolution. It also means that at increasingly higher magnetic fields, the advantages of extra sensitivity and increased ^1^H chemical shift resolution are not negated by the increased ^13^C increments needed in in order to resolve J-couplings. Enhancement of the splitting due to J-coupling can be achieved by incrementing the spin echo delay after the period where chemical shift of ^13^C evolves in parallel with the chemical shift evolution ( Figure 3). This spin echo refocuses the ^13^C chemical shift and the ^1^H- ^13^C coupling, but allows the ^13^C- ^13^C coupling to evolve further. The delays in the spin echo are proportional to those in the Ω+J _CC_ evolution period with the amount of extra coupling achieved being defined by the stretch of the J _CC_ increment compared to the Ω+J _CC_ increment. Thus, the ^13^C- ^13^C J-couplings can be expanded as required ( Figure 4). The ability to scale the signal splittings to varying extents means that the experiment can be tuned to the requirements of the sample and which metabolites are present, and of interest. Figure 4 demonstrates the effect of J-coupling splitting enhancement on 2D HSQC spectra, displaying C(6) of ^13^C enriched glucose. The ^13^C trace through the left-most signal ( Figure 4D), demonstrates clearly that while the singlet in the middle of the multiplet does not change, the splitting due to the ^1^J _CC_ coupling increases and in fact splits into multiple signals as the splittings of previously unresolved long-range couplings are amplified so that they are large enough to become resolved in the spectrum. These splittings can easily be simulated ( Figure 4E), thereby providing additional information with which to model metabolic pathways.

**Figure 5.**
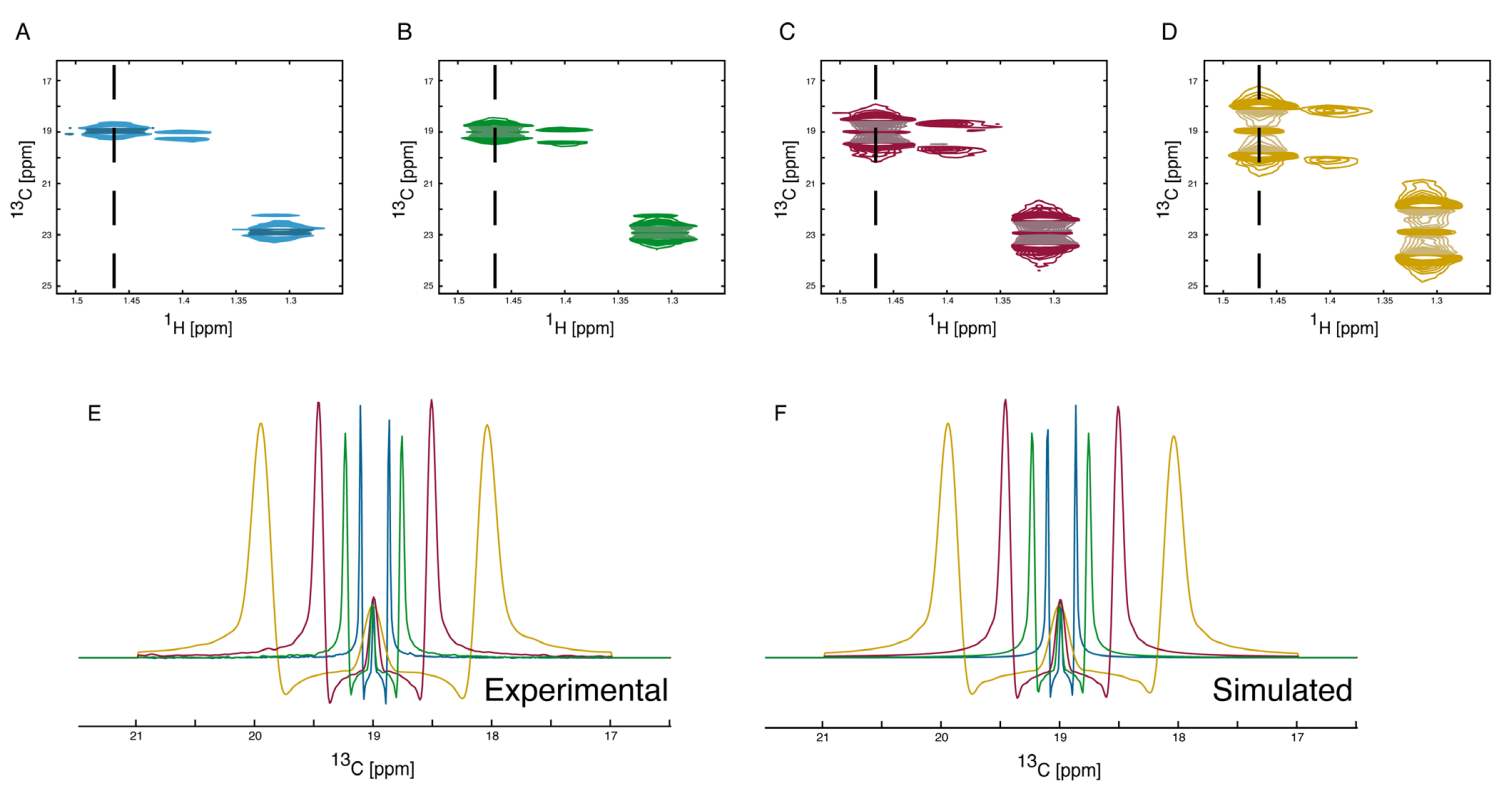
Simulation of J-coupling splitting enhanced spectra. ^1^H, ^13^C HSQC spectra showing the C(3) of alanine are shown ( **A**, **B**, **C** and **D**). The enhancement of J-coupling in the ^1^H, ^13^C-HSQC spectra leads to increased separation of coupled peaks and allows the collection of data with reduced numbers of increments in the ^13^C dimension resulting in shorter acquisition times. The number of points collected in the ^13^C dimension can be reduced to match the increase in J-coupling enhancement as the loss of resolution will be counteracted by the increase in separation of the coupled peaks. Spectra with no enhancement ( **A**), two-fold enhancement ( **B**), four-fold enhancement ( **C**) and eight-fold enhancement ( **D**) were collected. The overlay of the differently enhanced spectra clearly shows the effect of the enhancement ( **E**). The enhancement can be tailored to meet the need in order to maximise separation without significantly increasing signal overlap whilst achieving the maximum reduction in acquisition time possible. The splitting enhancement can easily be included in the simulation parameters ( **F**) resulting in no adverse effects on the simulated spectra. The simulation gives the following incorporation percentages. From the no J-coupling splitting enhancement spectrum 12.1% / 87.9% for [3- ^13^C] / [2,3- ^13^C], from the two-fold J-coupling splitting enhancement 12.0% / 88.0% for [3- ^13^C] / [2,3- ^13^C], from the four-fold J-coupling splitting enhancement 12.0% / 88.0% for [3- ^13^C] / [2,3- ^13^C] and from the eight-fold J-coupling splitting enhancement 12.1% / 87.9% for [3- ^13^C] / [2,3- ^13^C].

Large expansion of J-coupling also allows for rapid collection of data, as the resolution required to resolve them becomes diminished ( Figure 5). However, this should be tempered by the need to avoid unnecessary overlap of signals. To date, the authors have acquired 2D spectra with up to eight fold enhanced ^13^C J-couplings, combined with shortening the acquisition by using variable pulse sequence repetition times ^34^, leading to an overall decrease in acquisition time by a factor larger than 10 ( Figure 5). Panels A to D show the spectral region of the methyl groups of lactate and alanine. Panel E shows a cross section, as marked in the 2D spectra, from alanine, whereas panel F shows the corresponding simulations of those multiplets. The acquisition times for the different spectra were 233, 110, 51 and 24 minutes ( Table 1). Whilst the lines in the spectrum become broader due to the shorter acquisition times, this is negated by the increase in splittings, allowing the analysis of the multiplets with similar precision. Shorter acquisition times may be achieved by including spectral folding in the acquisition protocol or by incorporating new fast acquisition schemes such as ASAP- or ALSOFAST-HSQC ^35^. 

**Table 1.** Comparison of spectroscopic techniques. The acquisition time and signal to noise ratios of various experiments used in this study. A good signal to noise ratio can be achieved using the spectral filtering allowing rapid measurement of quantitative spectra. The signal to noise benefit of the HSQC over the 1D ^13^C acquisition is clearly seen. The effect of increasing the J-coupling splitting enhancement whilst simultaneously reducing the number of increments on the acquisition time of ^1^H, ^13^C-HSQC spectra is also shown.

Experiment	Acquisition time [mins]	Signal to noise ratio (CH _3_ of lactate)	Transients	TD	SW [ppm]	Splitting enhancement
^1^H 1D (all ^1^H)	2	327.31	8	16384	12	1
^1^H 1D ( ^13^C bound ^1^H)	2	66.13	8	16384	12	1
^1^H, ^13^C-HSQC	233	823.86	2	1024/8192	12/160	1
^1^H, ^13^C-HSQC	110	770.98	2	1024/4096	12/160	2
^1^H, ^13^C-HSQC	51	426.41	2	1024/2048	12/160	4
^1^H, ^13^C-HSQC	24	283.38	2	1024/1024	12/160	8
^13^C 1D (30° excitation)	1414	59.95	16384	65538	239	1

### ^15^N tracing

***Experimental setup.*** *2D ^14^N spectral filter* - Sample preparation is described elsewhere ^16^. 2D ^1^H ^13^C-HSQC NMR spectra with and without ^14^N filtering were acquired using a Bruker Avance III 600 MHz NMR spectrometer equipped with a 1.7 mm z-PFG TCI Cryoprobe. The HSQC spectra were acquired using 2 transients per increment with echo/anti-echo gradient coherence selection and an additional pre-saturation during the 1.5 s interscan relaxation delay to suppress the water resonance. The ^1^H dimension was acquired with a spectral width of 13 ppm using 512 complex data points. The ^13^C dimension was acquired with a spectral width of 160 ppm using 2048 data points. The spectra were processed with quadratic cosine window functions and without baseline correction to avoid complications in the multiplet analysis procedure.

*^13^C, ^15^N J-coupling splitting enhancement.* The human Renal Proximal tubule cell line (RPTEC/TERT1, supplied by Evercyte GmBH, Austria) was used to investigate the metabolic fates of both carbon from glucose, and carbon and nitrogen from glutamine. Cells were expanded as described elsewhere ^36^, with population doubling level (PDL) routinely tracked using in-house software (PDL calculator, EcoCyto). Cells with PDL between 43 and 45 were collated and seeded at a density of 4×10 ^4^/cm ^2 ^in 75 cm ^2^ flasks containing 240 µl/cm ^2^ flux media (Zenbio, cat DMEMf12-NGG002), supplemented as above with the addition of 17.5 mM [1,2- ^13^C] D-Glucose (sigma 453188) and 2 mM [U- ^13^C,U- ^15^N] L-Glutamine (sigma, 607983). Cell culture was continued for 48 hours to allow isotopic labelling, after which cells were washed with ice-cold saline solution (0.9%) and collected by scraping into 2 ml pre-chilled methanol (-20°C), 2 ml water (4°C) and 2 ml chloroform (-20°C). The solution was vigorously mixed for 10 minutes, after which lysates were centrifuged at 15,000 *g* for 15 min at 4°C. 1 ml of the sample was aspirated for NMR analysis. Samples were dried using a Savant (SPD1010) speedvac concentrator and then resuspended in 60 µL of 100 mM sodium phosphate buffer, containing 0.5 mM DSS, 2 mM Imidazole in D _2_O, pH 7.0. The samples were vortexed and subsequently sonicated for 10 min and then centrifuged at 15000 *g* for 30 seconds to collate the fluid. Finally 35 µl of the samples were transferred to 1.7 mm NMR tubes.

2D- ^1^H, ^13^C-HSQC and 2D- ^1^H, ^15^N-HSQC NMR spectra were acquired using a Bruker Avance III 600 MHz NMR spectrometer equipped with a 1.7 mm z-PFG TCI Cryoprobe. The HSQC spectra were acquired using 2 transients per increment with echo/anti-echo gradient coherence selection with an additional pre-saturation to suppress the water resonance during the 1.5 s interscan relaxation delay. The ^1^H dimension of the ^1^H, ^13^C-HSQC spectra was acquired with a spectral width of 12 ppm using 512 complex data points. The ^13^C dimension was acquired with a spectral width of 160 ppm using 25% (2048) of 8192 data points using a non-uniform sampling scheme. The ^1^H dimension of the ^1^H, ^15^N-HSQC spectra was acquired with a spectral width of 12 ppm using 1024 complex data points. The ^15^N dimension was acquired with a spectral width of 40 ppm using 256 data. All non-uniformly sampled spectra were reconstructed via the compressed sensing algorithm within MDDNMR (version 2.5) ^29^ and processed using NMRpipe (version 9.2) ^30^. All spectra were processed without baseline correction to avoid complications in the multiplet analysis procedure especially with regards to the negative peaks caused by the echo/anti-echo coherence selection with gradients.

All NMR spectra in this article were processed within the MetaboLab software package (version 2018. 07182055; http://metabolab.uk) ^22^.

***NMR methodology.*** Both aforementioned methods can be used to detect ^15^N labelling in metabolites, which alongside ^13^C isotope incorporation can provide additional much-needed information on the overlapping activity of multiple metabolic pathways. 2D spectroscopic filters are an extension of the 1D concept and as such can be used to simplify increasingly complex 2D spectra by selectively observing a subset of metabolites in which nuclei of interest have been incorporated. For example, the analysis of the ^13^C nuclei that are adjacent to ^15^N nuclei using 2D spectra permits a simplified unequivocal description of the nature in which two metabolic pathways converge.

**Figure 6.**
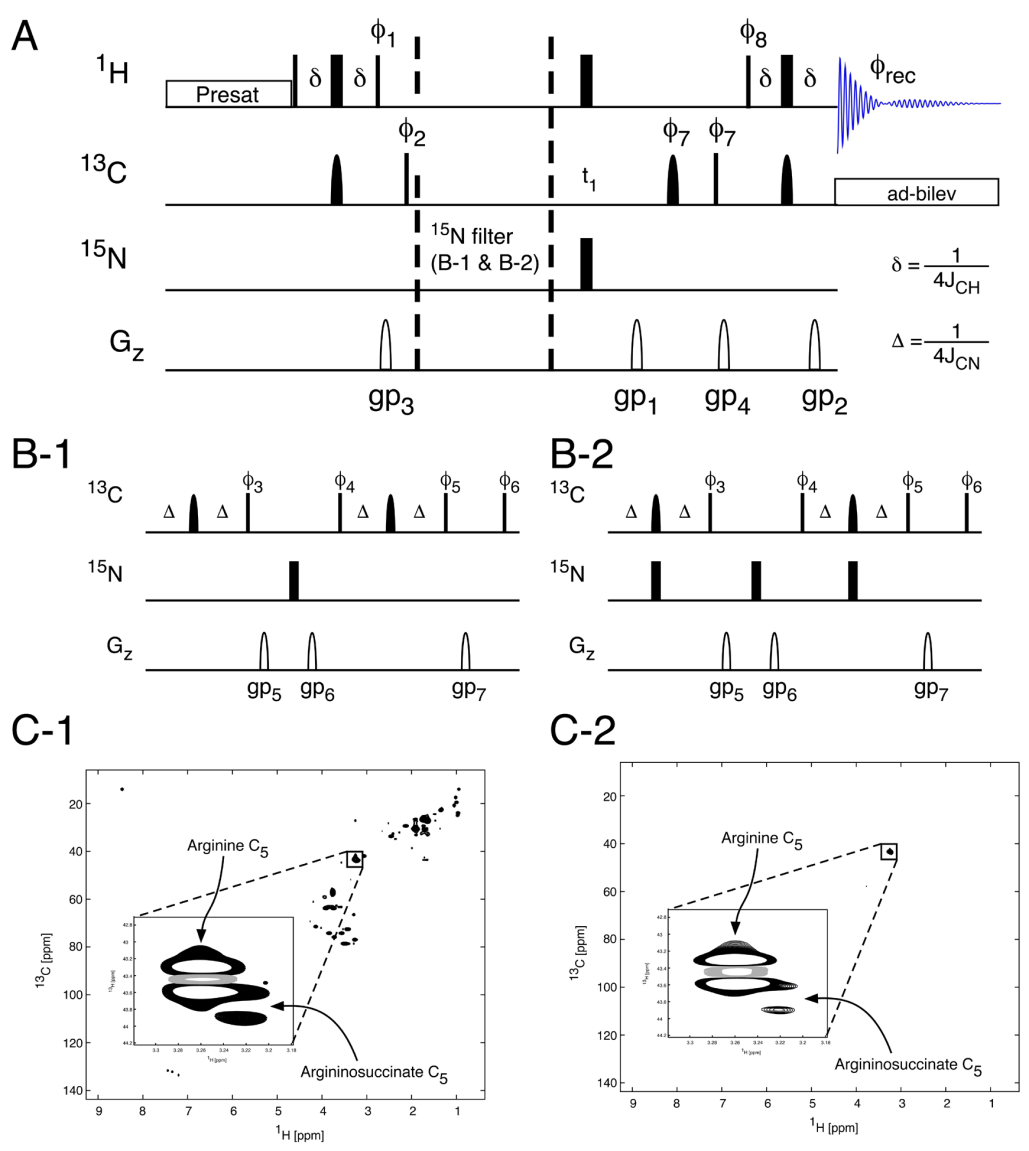
Filtered HSQC spectroscopy. The application of a ^15^N filtering block in the ^1^H, ^13^C-HSQC pulse sequence ( **A**) allows the observation of ^1^H, ^13^C groups directly coupled to ^15^N nuclei. In the sequence in panel **B-1** no filtering will be observed and the resulting spectrum ( **C-1**) will contain all ^1^H, ^13^C groups adjacent to either ^14^N or ^15^N nuclei. The use of a ^15^N filter ( **B-2**) will result in only those ^1^H, ^13^C groups adjacent to a ^15^N nuclei being observed in the resulting ^1^H, ^13^C HSQC spectrum ( **C2**). ^1^H, ^13^C J-coupling is suppressed during acquisition using adiabatic bilevel decoupling (ad-bilev) ^21^. The pulse phases are: ϕ _1_ = y; ϕ _2_ = x, -x; ϕ _3_ = x for the no filter sequence (panel **B-1**) and y for the ^15^N filtered sequence (panel **B-2**); ϕ _4_ = y, –y for the no filter sequence and y, y for the ^15^N filtered sequence; ϕ _5_ = y, -y for the no filter sequence x, -x for the ^15^N filtered sequence; ϕ _6_ = x, x, -x, -x; ϕ _7_ = x, x, x, x, -x, -x, -x, -x; ϕ _8_ = x, x, x, x, y, y, y, y; ϕ _rec_ = x, -x, -x, x, y, -y, -y, y.

Similar to the 1D method, the acquisition of two spectra is required in order to enable a quantitative analysis of the amount of ^15^N labelling in the presence of ^13^C labelling within the metabolite. If spectral simplification is the goal, a single spectrum is sufficient ^16^. The pulse sequence ( Figure 6) is a gradient selected ^1^H, ^13^C-HSQC spectrum with the spectral filter added once the ^1^H magnetisation has been transferred to the ^13^C nucleus. The spectrum collected with the ^14^N spectral filter (Panel C-2, Figure 6) contains only two visible NMR signals, corresponding to arginine and arginosuccinate, clearly showing how the filter can simplify complex spectra for easier analysis.

**Figure 7.**
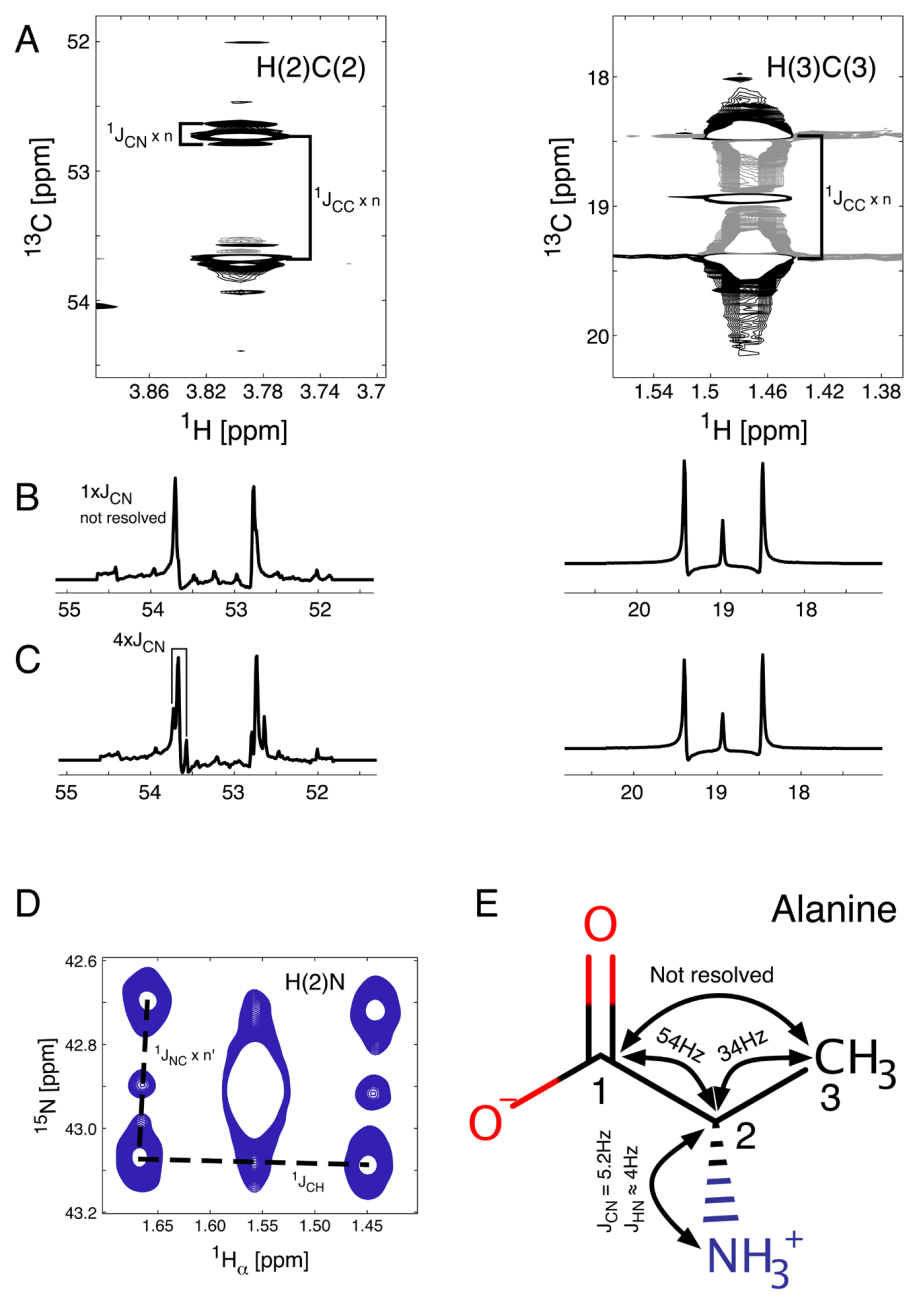
Splitting due to ^15^N and ^13^C incorporation. Regions of the ^1^H, ^13^C-HSQC spectrum containing signals from alanine (panels **A** & **B**). The ^13^C traces of the alanine signals are shown with either no J _CN_-coupling splitting enhancement ( **B**) or four-fold J _CN_-coupling splitting enhancement ( **C**). The signals are split by either ^1^J _CC_ or ^1^J _CN_ coupling contributions. The long range ^1^H, ^15^N-HSQC spectrum ( **D**) is composed of unlabelled alanine (central peak) and ^13^C/ ^15^N labelled alanine (6 outer signals, 4 split by ^2^J _HC_ and ^1^J _NC_ couplings and 2 only split by ^1^J _HC_ (values for the coupling constants are shown in panel **E**)).

While 2D spectral filters serve a purpose, their quantitative usage is limited by the variability of the ^1^J _CN_ constant. J-coupling splitting enhancement on the other hand can be easily extended to include ^13^C- ^15^N J- coupling splitting enhancement. Indeed, the addition of a single ^15^N π–pulse simultaneous with the central ^13^C π–pulse ( Figure 3) is sufficient to enhance the apparent ^13^C- ^15^N J-coupling splitting in ^1^H, ^13^C-HSQC spectra, an example of which is given in Figure 7. The 2D signals for the J _CN_ splitting scaled spectrum are shown in panel A. Panels B and C show traces of the ^13^C multiplets for carbon atoms 2 and 3 of alanine. While the J _CC_ splittings are enhanced by a factor of 4 in both spectra, the apparent J _CN_ splittings are unchanged in the spectrum in panel B, whereas they are enhanced by a factor of 4 in the spectra in panel C. Because the ^2^J _CN_ coupling between C(3) and N is negligible, both traces for C(3) overlap perfectly. C(2) on the other hand experiences a ^1^J _CN_ coupling, which is too small to be resolved when the J _CN_ splitting is not enhanced and is only detectable in panel C.

J _CN_ coupling, as any J-coupling, works in two directions, therefore a similar approach can be followed from the opposite direction. While amine groups of small molecules are notoriously difficult to observe due to chemical exchange of amine protons with solvent molecules, a long-range HSQC spectrum can be acquired. In such a spectrum proton magnetisation is transferred from H _α_ (the proton bound to C(2)) via the ^2^J _HN_ coupling. The splitting due to the J _NC_ coupling then can be enhanced to show the appearance of ^13^C labelling in molecules which contain ^15^N next to those labelled carbon nuclei. As in this case, where the ^13^C nucleus is also bound to the proton determining the chemical shift on the horizontal axis, that same proton signal will be split by the ^1^J _CH_ coupling constant. The result in this case is therefore a signal split into 7 2D components ( Figure 7, panel D), demonstrating that alanine was either recycled from unlabelled alanine which was incorporated into proteins, synthesised *de-novo* from [U- ^13^C] glucose and ^15^N labelled glutamate which originated from [U- ^13^C, U- ^15^N] glutamine that was added to the growth medium in addition to the [U- ^13^C] glucose or just synthesised *de novo* from [U- ^13^C] glucose with an unlabelled amino group transferred to form alanine. In conjunction with MS data, this complementarity between the 2D- ^1^H, ^13^C- and the 2D- ^1^H, ^15^N-HSQC spectra enables a model-free metabolism analysis using multiple nutrients as tracer sources in a single sample.

## Discussion

Changes in metabolism are increasingly being recognised as central to the pathogenesis of a number of different diseases. Although metabolomic studies have helped determine aspects of disease phenotype, tracing the changing use of specific metabolic pathways using stable isotope-enriched nutrients provides higher resolution information on altered metabolic pathway activity that may lead to the identification of specific novel therapeutic targets. Over the last few years, development of magnet and probe technology, including innovative ultra-sensitive microprobes, has enabled the study of systems that were not previously amenable to NMR spectroscopy. Parallel advancement in the methods used to acquire and analyse data from samples will increase the amount of information we can gain from such samples.

In this paper, we describe how spectral filters and J-splitting enhancement can be used in tracer-based metabolism studies. These techniques overcome some of the major hurdles in the use of NMR spectroscopy. A challenge in the analysis of NMR HSQC spectroscopy data has been the need for an additional “unlabelled” sample in order to determine absolute per carbon ^13^C incorporation percentages. However, samples cannot be assumed to be biologically identical, thus making analyses problematic due to the inability to determine accurate ^13^C isotope incorporation values. Systems that demonstrate greater inter-sample variation, such as *in vivo* tracer studies, are even more prone to these analytical issues. The use of spectral filters negates the requirement for two samples and instead a single sample can be used to determine absolute percentage ^13^C incorporation and thus allow the scaling of multiplets.

The 2D HSQC spectrum is a powerful tool in the study of metabolism as it takes advantage of the increased sensitivity of the ^1^H nucleus over ^13^C and using the splitting due to J-coupling in the ^13^C dimension allows the indirect visualisation of the ^13^C incorporation into quaternary carbons. However, long acquisitions times, even when using the latest NUS techniques, limits the number of samples that can be acquired. Reducing the experimental time makes the use of HSQC spectra a much more attractive method in the study of tracer-based metabolism. The use of echo/anti-echo for quadrature detection ensures efficient elimination of unwanted artefacts, whilst using only two scans per increment in the indirect dimension. The changes observed in line shape due to the quadrature detection are predictable and can be easily incorporated into line fitting analysis. As described elsewhere ^19^, the simulation procedure assumes weak coupling between the different carbon nuclei. The simulation is implemented as a spin echo before the acquisition of a ^13^C-FID to allow the evolution of ^13^C, ^13^C spin coupling prior to the first increment.

The ability to scale the visualised splittings due to J-coupling allows HSQC spectra to be acquired in time equivalent to that of a 1D ^1^H spectrum, but the HSQC spectrum contains significantly more information. The reduced time required to acquire HSQC spectra means that it is feasible to apply 2D methods to *in vivo* tracer-based metabolism studies, as well as allowing the use of greater sensitivity of higher field spectrometers while avoiding longer experiment times ( Table 1). Expansion of the splitting due to J-coupling can also bring out smaller long-range couplings that were not apparent in a normal HSQC spectrum. Thus, the scaling of splittings can either be used to decrease acquisition times by allowing data collection at lower resolution or to bring out smaller couplings not previously visible. These small couplings include the ^1^J _CN_ couplings that are found in many metabolites after the addition of metabolites labelled ^15^N in conjugation with ^13^C. This increases the information available and allows more in-depth analysis of complex metabolic pathways. In the example shown ( Figure 6), the cells used for this experiment were deficient in the expression of fumarate hydratase ^16^ and therefore contained high fumarate levels. One hypothesis was that argininosuccinate is synthesised from fumarate and arginine to minimise intracellular fumarate. In order to ascertain the signal assignment, we used [U- ^13^C, U- ^15^N] arginine and were able to show that ^15^N labelled argininosuccinate was being produced in the cells containing the knock out, but not in wild-type cells ^16^. This shows the utility of using multiple labelled nutrients to answer fundamental questions in metabolism.

In summary, the spectroscopic tools presented here open up new avenues for tracer-based metabolism studies. Scaling of signal splittings due to J-coupling leads to faster data collection of samples supplemented with nutrients enriched in stable isotopes, such as ^13^C and ^15^N. This enables profiling of metabolic pathways and can also be used to enhance sensitivity beyond current technical developments whilst maintaining reasonable data acquisition times. Ultimately, the use of 1D spectral filters as well as the fast acquisition of HSQC spectra leads to the possibility of tracing metabolism in real-time. In addition, simultaneous tracing with multiple nutrients will lead to unprecedented insight into the interplay of converging and intersecting metabolic pathways, both *in vitro* and *in vivo* ^37^.

## Data availability

All experimental data for this article is available at: http://doi.org/10.17605/OSF.IO/EQHN3 ^38^.

Experimental NMR datasets for HS-TrAM: ^12^C filtered (subdirectory 2) ^1^H spectrum, both of which are ^13^C decoupled during acquisition. Subdirectories 3 and 4 contain an unfiltered (3) and a ^12^C filtered (4) spectrum, both without ^13^C decoupling during acquisition. Subdirectories 5 and 6 contain POCE spectra, either with ^12^C- and ^13^C-bound ^1^H with same phase (subdirectory 5), or with opposite phase (subdirectory 6), both without ^13^C decoupling during acquisition. Subdirectory 7 contains a 30 degree excitation 1D ^13^C spectrum, acquired using a TXO Cryoprobe. The file jEnhanced_13C_HSQC.zip contains the ^1^H, ^13^C-HSQC spectra with different J-coupling splitting enhancements.

The file 13C_HSQC_14N_filter_and_15N_HSQC.zip contains an unfiltered (subdirectory 1) and a ^14^N filtered (subdirectory 2) ^1^H, ^13^C-HSQC spectrum. The file jEnhanced_13C_15N_HSQC.zip contains the following spectra: 4 x ^13^C, ^13^C splitting enhancement ^1^H, ^13^C-HSQC in subdirectory 1, 4 x ^13^C, ^13^C splitting enhancement and 4 x ^13^C, ^15^N splitting enhancement ^1^H, ^13^C-HSQC in subdirectory 2 and 4 x ^13^C, ^15^N splitting enhancement ^1^H, ^15^N-HSQC in subdirectory 3.

License: CC0 1.0 Universal
