# Supplementary material for: High-Speed Tracer Analysis of Metabolism (HS-TrAM)
Source: Wellcome Open Res. 2018 Aug 22;3:5. Originally published 2018 Jan 12. [Version 2] doi: 10.12688/wellcomeopenres.13387.2 (PMC5811808; doi:10.12688/wellcomeopenres.13387.2)
Supplement: Peer review history

# Peer review: Referee response for version 2

**DOI:** 10.21956/wellcomeopenres.16084.r33749

**Published:** 2018-09-19

**Authors:** Paul C. Driscoll

**Affiliations:** 1 Francis Crick Institute, London, UK

The revised manuscript has been significantly improved compared to the original version, and give an enhanced prospect that other researchers can attempt to employ the new approach to measurement of stable isotope enrichment in biological samples.

 I think it worth pointing out the following:

 It is not made clear in the figure legend for Figure 2 (though it is mentioned in the main text) that the data in panels A-3 and B-3 were obtained in the absence of 13C-decoupling.

 Page 6: The authors refer to the invariance of the spectral filter with respect to differential relaxation 1H rates. This comment appears to refer to chemically distinct 1H atoms.  It seems entirely probable that the relaxation  rate of chemically equivalent 12C- and 13C-bound 1H nuclei would be non-identical. Such differences would introduce a systematic difference in the signal response for the different (X-enriched/X-nonenriched) sets of molecules and therefore lead to a (perhaps small) error in the derived level of X-enrichment - particularly when the X-enrichment is low. 

 The performance of 13C-pulses and 13C-decoupling may be 13-C offset -dependent. The paper contains no demonstration that the choice of offset leads to any systematic error in the derived 13C-enrichments.

 Figure 6 legend: presumably the spectrum in C-1 also contained 1H/!3C-signals from CH group unattached to any nitrogen atom. 

 Figure 7D: The 1H chemical shift scale does not appear to be consistent with the 1Halpha NMR resonances.

 Overall, the paper would be improved by further copyediting. On panel C-2 of Figure 6 is directly referred to in the main text. I note numerous non-sequiturs, inappropriate letter cases (e.g. page 7: D-Glucose); inappropriate use of numerals instead of number words (e.g. Figure 7 legend (better: six outer signals, four split...)

I have read this submission. I believe that I have an appropriate level of expertise to confirm that it is of an acceptable scientific standard.

# Peer review: Referee response for version 2

**DOI:** 10.21956/wellcomeopenres.16084.r33747

**Published:** 2018-09-03

**Authors:** Hector C. Keun

**Affiliations:** 1 Department of Surgery and Cancer, Faculty of Medicine, Imperial College London, London, UK

The revised version submitted is certainly much improved with greater clarity in the presentation. I'm not sure that all suggestions have been addressed, particularly with respect to comparison and justification versus other approaches.  There is still no validation with known levels of enrichment.  However I think there is sufficient detail in the manuscript to understand and repeat the experimental set up and thus for the sequences proposed to be evaluated in more complete way.  Hence  the work should be indexed pending the minor corrections below.

 Minor corrections:

 In figure 2 panels A-3/B-3: these should be labelled on figure and it should be stated in legend that the 13C decoupling during acquisition has been switched off.  The reader only gets this from the main text and it is confusing.

 Given that figure 2 suggests that the authors have the same samples with/without decoupling, then they can report the estimation of 13C enrichment from the DSS peak presented using their method and the differences in 13C enrichment estimates of lactate from the decoupled and coupled spectra.

I have read this submission. I believe that I have an appropriate level of expertise to confirm that it is of an acceptable scientific standard.

# Peer review: Referee response for version 2

**DOI:** 10.21956/wellcomeopenres.16084.r33748

**Published:** 2018-08-23

**Authors:** Sebastien Serres

**Affiliations:** 1 University of Nottingham, Nottingham, UK

This is a much improved version that fully covers all the points I mentioned in my previous report. 

 Simplifying the biological model has helped to get the message across. The utility of the method is now evident and mislabeling in figure and legends have been corrected.

 I am happy with these corrections and approve indexing of this paper.

I have read this submission. I believe that I have an appropriate level of expertise to confirm that it is of an acceptable scientific standard.

# Peer review: Referee response for version 1

**DOI:** 10.21956/wellcomeopenres.14535.r29776

**Published:** 2018-03-23

**Authors:** Hector C. Keun

**Affiliations:** 1 Department of Surgery and Cancer, Faculty of Medicine, Imperial College London, London, UK

In this manuscript the authors present a set of novel experimental schemes designed to reduce the time and experimental complexity of measuring isotope enrichment in biological samples.   They present a filter designed to allow quantitative analysis of enrichment without an unlabelled reference sample, and then expand to include J-scaling to reduce experiment time and present a 15N filtered 1H-13C HSQC to monitor labile 15N via JNC.  The methods could be very useful for many isotope-labelling experiments, increasing the confidence and ease with which various features can be extracted from such experiments.

 Main points:

  

- The authors state that the new filter presented will negate the requirement for a matched unlabelled sample to calculate isotopic enrichment.  It would be really helpful to see that explicitly demonstrated (could be with simulated mixtures of 15N/13C/unlabelled substrates perhaps spiked into a relevant background, not necessarily a real labelling expt in a biological system) and the performance compared to more conventional experimental approaches e.g. 1D 13C with proton decoupling,  or indeed mass spec?  Would be particularly useful to know the minimum amounts of fractional enrichment that can be identified.

 

- J-(up)scaling has been reported before, implemented in different ways (e.g. Furihata & Tashiro MRC 2013 ^1^, Willker et al. JMR 1997 ^2^ and refs within, several by Zangger et al).  It would be good to cite these instances and highlight what is different about the authors’ scheme. 

 

- While the J-scaling approach clearly ought to help resolution and quantitative analysis of overlapping multiplets from different isotopomers,  again it would be good to see some complete analysis to that effect and a comparison to the standard 2D experiments or other J-scaling alternatives.

 

- As much of the utility of the method for its stated purpose (isotopomer analysis) is contingent on the peak fitting algorithms used, some further details on this should be provided.

 

- In general there are lot of apparent typos with respect to subscript/superscript fonts, mislabelling in figures/legends – I presume something went wrong with formatting into final form, the authors should check through thoroughly.

 

- Some better annotation / commenting of the pulse sequence in the main text would be useful (gradient pulses used, necessary additional delays etc).

 

- The blending of methods and results leads to some confusion when going through figures.  In general these are not annotated such that the reader understands what the sample in question is, nor why the analysis is presented thus.  It can be inferred from the main text but is not always made explicit.  For example in Figure 2 one presumes that we are shown resonances where there is believed to be enrichment e.g. lactate, but others where there is not (and are scaled by 100 to demonstrate this).  I think the Figure legends and text should be more explicit.

 

 Minor points:

 Figure 2.  “gp1 and gp2”? Not labelled. “Chirp pulses with yB1max = ?? Hz”?

 Figure 5: “13H ppm”? Also does yellow indicate negative intensity?

 P8. “D20”? “1.

 Figure 6.  Need to label couplings on the molecule for clarity

I have read this submission. I believe that I have an appropriate level of expertise to confirm that it is of an acceptable scientific standard, however I have significant reservations, as outlined above.

## References

[1] FurihataKTashiroM: BASHD-J-resolved-HMBC, an efficient method for measuring proton-proton and heteronuclear long-range coupling constants. *Magn Reson Chem*.52(1-2) : 10.1002/mrc.4032 27-31 10.1002/mrc.4032 24282023

[2] WillkerWFlögelULeibfritzD: Ultra-high-resolved HSQC spectra of multiple- 13C-labeled biofluids. *J Magn Reson*.1997;125(1) : 10.1006/jmre.1996.1101 216-9 10.1006/jmre.1996.1101 9245385

# Response

**Published:** 2018-08-17

**Authors:** Christian Ludwig

**Affiliations:** University of Birmingham, UK

We thank Hector Keun for his comments and suggestions. These have been very helpful to improve the quality of the manuscript.

 Reviewer: The authors state that the new filter presented will negate the requirement for a matched unlabelled sample to calculate isotopic enrichment. It would be really helpful to see that explicitly demonstrated (could be with simulated mixtures of 15N/13C/unlabelled substrates perhaps spiked into a relevant background, not necessarily a real labelling expt in a biological system) and the performance compared to more conventional experimental approaches e.g. 1D 13C with proton decoupling, or indeed mass spec? Would be particularly useful to know the minimum amounts of fractional enrichment that can be identified. 

**Response: We included spectra without ^13^C, ^1^H decoupling demonstrating not only the quality of the ^12^CH _3_signal removal from the spectrum, but also the linearity of the intensities between the spectra, as the satellites in both spectra appear at 0.5% intensity of the ^12^CH _3_signal. This can also serve as an indication for the minimum of fractional enrichment possible to identify. If possible, we prefer to acquire mass spec data. However, this may not always be possible, e.g. when analysing samples of intact tissue.**

 Reviewer: *J-(up)scaling has been reported before, implemented in different ways (e.g. Furihata & Tashiro MRC 2013* *1* *, Willker et al. JMR 1997* *2* *and refs within, several by Zangger et al). It would be good to cite these instances and highlight what is different about the authors’ scheme.*

**Response: We included those references in the main text.**

 Reviewer: While the J-scaling approach clearly ought to help resolution and quantitative analysis of overlapping multiplets from different isotopomers, again it would be good to see some complete analysis to that effect and a comparison to the standard 2D experiments or other J-scaling alternatives. 

 As much of the utility of the method for its stated purpose (isotopomer analysis) is contingent on the peak fitting algorithms used, some further details on this should be provided. 

**Response: We included complete multiplet analyses for splitting scaling with enhanced resolution for small couplings (figure 4) as well as a series of 2D spectra demonstrating the potential to accelerate acquisition times (figure 5).**

 Reviewer: In general there are lot of apparent typos with respect to subscript/superscript fonts, mislabelling in figures/legends – I presume something went wrong with formatting into final form, the authors should check through thoroughly. 

**Response: We have checked thoroughly.**

 Reviewer: Some better annotation / commenting of the pulse sequence in the main text would be useful (gradient pulses used, necessary additional delays etc). 

**Response: We redesigned most of the figures and enhanced the annotation of the pulse sequences.**

 Reviewer: The blending of methods and results leads to some confusion when going through figures. In general these are not annotated such that the reader understands what the sample in question is, nor why the analysis is presented thus. It can be inferred from the main text but is not always made explicit. For example in Figure 2 one presumes that we are shown resonances where there is believed to be enrichment e.g. lactate, but others where there is not (and are scaled by 100 to demonstrate this). I think the Figure legends and text should be more explicit. 

**Response: We redesigned most figures (including figure 2) and their legends and made references to the figures more explicit in the main text.**

 Reviewer: Figure 2. “gp1 and gp2”? Not labelled. “Chirp pulses with yB1max = ?? Hz”? Figure 5: “13H ppm”? 

**Response: All pulses are now properly labelled. We added the missing value for** **g** **B _1max_and changed ^13^H to ^13^C in figure 5**.

 Reviewer: Also does yellow indicate negative intensity?

**Response: We redesigned this figure and removed colour from the spectra plots, this should be clearer now.**

 Reviewer: P8. “D20”? “1.

**Response: All D20 occurences have been changed to D2O**

 Reviewer: Figure 6. Need to label couplings on the molecule for clarity 

**Response:** **We included all detectable couplings in the molecule plot.**

# Peer review: Referee response for version 1

**DOI:** 10.21956/wellcomeopenres.14535.r29775

**Published:** 2018-02-12

**Authors:** Julian L. Griffin

**Affiliations:** 1 Department of Biochemistry, Cambridge Systems Biology Centre, University of Cambridge, Cambridge, UK

Smith and colleagues describe a new approach for acquiring 13C NMR labelling data as part of flux based analyses of metabolism. There has been much recent interest in metabolomics and metabolism research in the application of stable isotope approaches to probe metabolite fluxes. While mass spectrometry is increasingly being used for these labelling experiments, this approach cannot readily determine the position of labelling, limiting the information that can be obtained – for example, different pathways can result in different labelling patterns of certain metabolites. While NMR spectroscopy can determine where labelling is in a given molecule, the approach has poor sensitivity and can take significant time to acquire the datasets. Smith and colleagues set out to improve one type of 2-dimensional NMR technique – a heteronuclear single quantum coherence (HSQC) spectroscopy – to reduce the time and increase the sensitivity of the approach, thus aiding those who use NMR spectroscopy for flux analysis. The manuscript is well written and the data and tools used available to the reader which should aid the dissemination of the products of this research. I particularly liked the application using 13C and 15N dual labelling to follow amino acid metabolism – both in terms of the carbon backbone and the ammine group of amino acids.  I do have some specific questions though that I would like the authors to address.

1. Abstract: Strictly speaking it should be NMR spectroscopy rather than simply NMR.
2. Abstract: What splitting are they referring to? Could they be more precise in the abstract?
3. Introduction: “We currently have limited knowledge about the compartmentalisation of metabolic pathways in metabolically-active organelles, such as mitochondria, and therefore whether the same metabolite is selectively utilised for distinct purposes in different compartments.” A good reference (or references) for the use of 13C NMR spectroscopy to examine metabolic compartmentation would be the work of E.D. Lewandowski.
4. Despite the fact that metabolites can arise from multiple sources, the contribution of the different metabolic pathways to the synthesis of this metabolite can be determined through the analysis of the ^13^C and/or ^15^N distribution within the metabolite.” This should be expanded on - with NMR spectroscopy we can observe exactly where the label is in a metabolite - this is exceptionally hard using mass spectrometry and probably open to a lot of confusion.
5. NMR methodology. It would be useful to have the approximate times for the various pulse sequences discussed so the reader can judge how long the spectra required to acquire.
6. NMR methodology. “The trace through the left-most signal, displayed in panel D, demonstrates clearly that while the singlet in the middle of the multiplet does not change, the apparent ^1^J _CC_ constant increases and in fact splits into multiple signals as previously unresolved long-range couplings are amplified so that they are large enough to become resolved in the spectrum.” Does this create problems for quantification if the signals are beginning to be split?
7. In Figure 2 why can no 13C satellites be seen around the 1H NMR doublet of lactate? Presumably I’m missing something as I note the 13C spectrum is uncoupled from 1H-13C interactions and we only “see” the doublet from 1H-1H couplings. Note the figure legend needs to discuss A and B in terms of labels.
8. Figure 4D. How easy is it to quantify from such complex line shapes as shown in Figure 4D?

I have read this submission. I believe that I have an appropriate level of expertise to confirm that it is of an acceptable scientific standard.

# Response

**Published:** 2018-08-17

**Authors:** Christian Ludwig

**Affiliations:** University of Birmingham, UK

We thank Julian Griffin for his comments and suggestions. These have been very helpful to improve the quality of the manuscript. 

 Reviewer: Smith and colleagues describe a new approach for acquiring 13C NMR labelling data as part of flux based analyses of metabolism. There has been much recent interest in metabolomics and metabolism research in the application of stable isotope approaches to probe metabolite fluxes. While mass spectrometry is increasingly being used for these labelling experiments, this approach cannot readily determine the position of labelling, limiting the information that can be obtained – for example, different pathways can result in different labelling patterns of certain metabolites. While NMR spectroscopy can determine where labelling is in a given molecule, the approach has poor sensitivity and can take significant time to acquire the datasets. Smith and colleagues set out to improve one type of 2-dimensional NMR technique – a heteronuclear single quantum coherence (HSQC) spectroscopy – to reduce the time and increase the sensitivity of the approach, thus aiding those who use NMR spectroscopy for flux analysis. The manuscript is well written and the data and tools used available to the reader which should aid the dissemination of the products of this research. I particularly liked the application using 13C and 15N dual labelling to follow amino acid metabolism – both in terms of the carbon backbone and the ammine group of amino acids. I do have some specific questions though that I would like the authors to address. 

1. Abstract: Strictly speaking it should be NMR spectroscopy rather than simply NMR. 

**Response:** **We changed NMR to NMR spectroscopy**

  

1. Reviewer: Abstract: What splitting are they referring to? Could they be more precise in the abstract?

**Response:** **We changed the description to be explicit about the homonuclear 13C,13C and heteronuclear 13C,15N J-couplings referred to in the abstract.**

  

1. Reviewer: Introduction: “We currently have limited knowledge about the compartmentalisation of metabolic pathways in metabolically-active organelles, such as mitochondria, and therefore whether the same metabolite is selectively utilised for distinct purposes in different compartments.” A good reference (or references) for the use of 13C NMR spectroscopy to examine metabolic compartmentation would be the work of E.D. Lewandowski. 

**Response:** **We added this reference**

  

1. Reviewer: Despite the fact that metabolites can arise from multiple sources, the contribution of the different metabolic pathways to the synthesis of this metabolite can be determined through the analysis of the 13C and/or 15N distribution within the metabolite.” This should be expanded on - with NMR spectroscopy we can observe exactly where the label is in a metabolite - this is exceptionally hard using mass spectrometry and probably open to a lot of confusion. 

**Response:** **We added a statement about the potential of NMR data.**

  

1. Reviewer: NMR methodology. It would be useful to have the approximate times for the various pulse sequences discussed so the reader can judge how long the spectra required to acquire. 

**Response:** **We added a table with acquisition times as well as pros and cons of the approaches.**

  

1. Reviewer: NMR methodology. “The trace through the left-most signal, displayed in panel D, demonstrates clearly that while the singlet in the middle of the multiplet does not change, the apparent 1JCC constant increases and in fact splits into multiple signals as previously unresolved long-range couplings are amplified so that they are large enough to become resolved in the spectrum.” Does this create problems for quantification if the signals are beginning to be split? 

**Response:** **We included a quantitative analysis with multiplet simulations, demonstrating that the multiplet compositions do not change with different amounts of scaling.**

  

1. Reviewer: In Figure 2 why can no 13C satellites be seen around the 1H NMR doublet of lactate? Presumably I’m missing something as I note the 13C spectrum is uncoupled from 1H-13C interactions and we only “see” the doublet from 1H-1H couplings. Note the figure legend needs to discuss A and B in terms of labels. 

**Response: We redesigned the figure and included a spectrum without ^13^C, ^1^H decoupling. This should make it easier to understand why there are no satellites around lactate.**

  

1. Reviewer: Figure 4D. How easy is it to quantify from such complex line shapes as shown in Figure 4D? 

**Response: We included a quantitative analysis including a line shape fit.**

# Peer review: Referee response for version 1

**DOI:** 10.21956/wellcomeopenres.14535.r29774

**Published:** 2018-01-23

**Authors:** Paul C. Driscoll

**Affiliations:** 1 Francis Crick Institute, London, UK

In this manuscript the authors have built on their own previous work wherein high resolution 2D ^1^H ^13^C HSQC NMR spectra were used together with sophisticated data-fitting tools to provide quantitative measures of isotope incorporation at single positions within metabolites. The specific procedures developed here significantly expand the scope of the method and in principle constitute a new paradigm in isotope tracer analysis. One aspect (and it is only one of several utilities that arise) is that the approach should enable real time measurements in isotopomer-based NMR metabolic profiling with a vastly higher time resolution than would otherwise be possible. A particularly advantageous aspect is the demonstration of the detection of adjacent ^13^C- ^15^N dual isotope labelling with enhanced sensitivity and precision, opening up the potential to simultaneously assess the metabolic flux of both carbon and nitrogen atoms.

 I have abundant enthusiasm for the ingenuity and potential of this work, and find very little that is technically questionable with the approach. However I did find several aspects where the manuscript should be enhanced or, indeed, corrected to make it more useful to the more- or less-informed reader.

 I found a number of aspects of the structure of the document require attention. Namely:

 Aspects of Methods are unnecessarily (and confusingly) mixed up with the Results, for example on pages 6-7 Biological and NMR Methods are completely jumbled.

 I do not think it is pedantry to say that J-couplings cannot be ‘enhanced’. The resonance _splittings_ due to [magnetization evolution due to] J-coupling can be enhanced, but J-coupling _constants_ are exactly that: constant. More care should be taken with the language used to describe the ‘J-scaling’ (itself strictly a misnomer in my opinion) concept employed. I note that the treatment assumes weak coupling between ^13^C atoms (well, in that the line shape is not described anywhere – or shown fitted here, I expect that assumption is behind the Metabolab treatment). It is not a bad assumption but this condition should be stated.

 Is ‘J-scaling’ truly ‘novel? I found reference to downscaling of J-couplings from the relatively early NMR literature, and wonder whether upscaling of J-splittings has been employed in the more recent field of residual dipolar coupling measurements.

 I would feel more comfortable for the less expert reader if the authors would comment on the extent to which, if at all, their two approaches might be sensitive to differential ^1^H relaxation rates, signal multiplicities and ^1^JCH coupling constants. I think the section on page 3 describing the adiabatic decoupling during the acquisition of the FID requires greater caveating than is provided. For example, one should check the manufacturer’s specification for the particular probe employed, and the authors here could indicate the average power dissipation and whether the cryogas heater current is above its lower limit during such a long period of RF irradiation. When 0.5 Hz line broadening apodization is being employed, do you need 2.25 s of acquisition?

 I do not understand why baseline correction (e.g. zeroth order to remove offsets) would interfere with data fitting, as mentioned more than once.

 The figure captions are mostly inadequate. Despite the figure panels being labelled A, B, C etc. the captions themselves do not refer to these labels. More detail is required here.

 I struggled with the description of the spectral filter experiment sketched in Figure 2 and described on page 3. To assist the less NMR-savvy reader, I would prefer to see this cast in terms of two different NMR experiments with separate depictions of the pulse sequence(s).

 Throughout the main text I would prefer not to see references to ‘the blue spectrum’, ‘the grey pulse’, but rather more explicit terms used.

 Figure 2A: see comment above. Labels for gp1 and gp2 are missing. ‘ad-bilev’ is not explained. Reference should be made to time gaps constituted by the gradient recovery delays.

 Figure 2B: it is not explicit what is the utility of scaling the blue spectrum by 100?

 Figure 3: The J-coupling is not enhanced (see above). The Evolution and Increment lines in the figure are (confusingly) not horizontally aligned. ‘n’ is not explained. Why is the ^13^C ‘J-scaling’ pulse shown in gray? Caption: ‘simultaneously with’ would be better than ‘parallel’.

 Figure 4: The cross-sectional vertical lines would be better in dashed format. Positive and negative contour colour differences are not explained. It might be worthwhile to comment on the asymmetric structure of the cross peak in the ^13^C dimension. Caption: there is no reference to A,B,C,D panel labels. The description of the pulse sequence scaling evolution delays needs to be improved (‘the J-scaling’ is shorthand; ‘the J-only evolution’ period might be better).

 Figure 5:  A and B labels for the different figure elements are missing. Why are some of the ^13^C pulses shown in grey? I would prefer it if the two NMR spectra were not superposed, but rather plotted side by side. Caption: It is ambiguous whether the grey ^15^N pulse is employed along with the amber pulses. I am not keen on the use of colour in the exposition of the phase cycles.

 Figure 6: Panel C seems to have more to do with A than B, so order is confusing. ‘n’, ‘n’’, ‘n’’’ ‘H[2]N’, ‘H[2]C[2]’ and ‘H[3]C[3]’ are not clearly explained (molecular structure needs numbering). The caption is in error when referencing B and C. Panel C switches from C[2] to C(2), etc. What do the different colour traces in panel C indicate? The caption does not explain. Note 1: on page 7 of the text there is reference to a panel D, and this strangle out of order as reference to panels A and B comes later. Note 2: Later the text describes the long range ^1^H ^15^N HSQC spectrum shown in panel C but without reference to it. It appears to describe the spectrum in terms of carbon C(2) and a two bond ^2^J _NH_ coupling, but should properly be C(3) (Cbeta) and ^2^J _NCbeta_. Note 3: The text goes on (page 8) to discuss the metabolic pathway in a section that would more properly be in the Discussion, and then there is a short paragraph about Metabolab that should be in the Methods section.

 Discussion:

 When 1D spectra are mentioned (page 11) is this meant to refer to ^13^C 1D spectra?

 There is a claim for ‘unprecedented’ detail. The utility of J-scaling to reveal small couplings is not very well exemplified by the results, since no fitted cross sections are shown. Also whilst real-time measurements of metabolism using this approach can be anticipated, no (even synthetic) example is shown. The ‘High Speed’ moniker in the title for the manuscript is not strongly justified by the data shown, and not strongly emphasized by the account given. Would it not be preferable to provide side-by-side demonstrations of the effective information content, for a given complex sample* in (i) a 1D ^1^H-decoupled ^13^C spectrum; (ii) a non-aliased 2D ^1^H ^13^C-HSQC spectrum with minimum phased cycle; (iii) an aliased version of (ii); and (iv) the ‘J-scaled’ versions of (ii) and (iii)?  * of arbitrary complexity and concentration?

 Throughout the manuscript, greater care is required for the use of numerals instead of words (where appropriate, e.g. two scans, no 2 scans); spaces between numbers and units; avoidance of unexplained abbreviations (e.g. WIT-15); appropriate use of spectra/spectrum; avoidance of ambiguous orphan pronouns (e.g. ‘this/it’ [verb]); anthropomorphization (e.g. ‘cells form…’ page 10); it is D _2_O, not D _2_0; almost invariably ‘spectrum’ should follow HSQC. I would be happy to proof read any revised version of the manuscript to help iron out any similar issues remaining.

 In summary, whilst I see great value in the ideas presented here, and hope to employ them in our own future work, I believe that the authors could substantially upgrade the description of the new approach in a revised version of the manuscript.

I have read this submission. I believe that I have an appropriate level of expertise to confirm that it is of an acceptable scientific standard, however I have significant reservations, as outlined above.

# Response

**Published:** 2018-08-17

**Authors:** Christian Ludwig

**Affiliations:** University of Birmingham, UK

We thank Paul Driscoll for his comments and suggestions. These have been very helpful to improve the quality of the manuscript.

 Reviewer: In this manuscript the authors have built on their own previous work wherein high resolution 2D 1H13C HSQC NMR spectra were used together with sophisticated data-fitting tools to provide quantitative 

 measures of isotope incorporation at single positions within metabolites. The specific procedures developed here significantly expand the scope of the method and in principle constitute a new paradigm in isotope tracer analysis. One aspect (and it is only one of several utilities that arise) is that the approach should enable real time measurements in isotopomer-based NMR metabolic profiling with a vastly higher time resolution than would otherwise be possible. A particularly advantageous aspect is the demonstration of the detection of adjacent 13C-15N dual isotope labelling with enhanced sensitivity and precision, opening up the potential to simultaneously assess the metabolic flux of both carbon and nitrogen atoms. 

 I have abundant enthusiasm for the ingenuity and potential of this work, and find very little that is technically questionable with the approach. However I did find several aspects where the manuscript should be enhanced or, indeed, corrected to make it more useful to the more- or less-informed reader. 

 I found a number of aspects of the structure of the document require attention. Namely:

 Aspects of Methods are unnecessarily (and confusingly) mixed up with the Results, for example on pages 6-7 Biological and NMR Methods are completely jumbled.

**Response:** **We separated the biological methods from the NMR methods.**

 Reviewer: I do not think it is pedantry to say that J-couplings cannot be ‘enhanced’. The resonance _splittings_ due to [magnetization evolution due to] J-coupling can be enhanced, but J-coupling _constants_ are exactly that: constant. More care should be taken with the language used to describe the ‘J-scaling’ (itself strictly a misnomer in my opinion) concept employed. I note that the treatment assumes weak coupling between 13C atoms (well, in that the line shape is not described anywhere – or shown fitted here, I expect that assumption is behind the Metabolab treatment). It is not a bad assumption but this condition should be stated. 

**Response:** **We changed the text to reflect that it is only the splittings that are enhanced in the spectra. We added more details on the line shape simulation and explicitly stated the weak coupling assumption behind the data analysis procedure.**

 Reviewer: Is ‘J-scaling’ truly ‘novel? I found reference to downscaling of J-couplings from the relatively early NMR literature, and wonder whether upscaling of J-splittings has been employed in the more recent field of residual dipolar coupling measurements. 

**Response:** **We added relevant citations.**

 Reviewer: I would feel more comfortable for the less expert reader if the authors would comment on the extent to which, if at all, their two approaches might be sensitive to differential 1H relaxation rates, signal multiplicities and 1JCH coupling constants. I think the section on page 3 describing the adiabatic decoupling during the acquisition of the FID requires greater caveating than is provided. For example, one should check the manufacturer’s specification for the particular probe employed, and the authors here could indicate the average power dissipation and whether the cryogas heater current is above its lower limit during such a long period of RF irradiation. When 0.5 Hz line broadening apodization is being employed, do you need 2.25 s of acquisition? 

**Response:** **We added more caveats and explanations addressing all those points.**

 Reviewer: I do not understand why baseline correction (e.g. zeroth order to remove offsets) would interfere with data fitting, as mentioned more than once. 

**Response:** **Although we didn’t try zeroth order baseline corrections, we don’t really need this as the baseline is centred around zero. Higher order baseline corrections will interefere with the negative aspects of the multiplets.**

 Reviewer: The figure captions are mostly inadequate. Despite the figure panels being labelled A, B, C etc. the captions themselves do not refer to these labels. More detail is required here. 

**Response:** **We redesigned most figures and have taken care to ensure that labels are referred to appropriately.**

 Reviewer: I struggled with the description of the spectral filter experiment sketched in Figure 2 and described on page 3. To assist the less NMR-savvy reader, I would prefer to see this cast in terms of two different NMR experiments with separate depictions of the pulse sequence(s). 

 Throughout the main text I would prefer not to see references to ‘the blue spectrum’, ‘the grey pulse’, but rather more explicit terms used. 

**Response:** **Figure 2 has been redesigned and all colour references have been removed. The explanation has been improved.**

 Reviewer: Figure 2A: see comment above. Labels for gp1 and gp2 are missing. ‘ad-bilev’ is not explained. Reference should be made to time gaps constituted by the gradient recovery delays. 

**Response:** **Figure 2 has been redesigned and all colour references have been removed. The explanation has been improved. ad-bilev is now explained and the reference has been added.**

 Reviewer: Figure 2B: it is not explicit what is the utility of scaling the blue spectrum by 100? 

**Response:** **This was done to demonstrate the ability to detect natural abundance of ^13^C. We understand that this could be confusing and have now removed the scaled spectrum and replaced it with a non-decoupled spectrum of DSS with its ^13^C satellites. We hope that the new figure is clearer.**

 Reviewer: Figure 3: The J-coupling is not enhanced (see above). The Evolution and Increment lines in the figure are (confusingly) not horizontally aligned. ‘n’ is not explained. Why is the 13C ‘J-scaling’ pulse shown in gray? Caption: ‘simultaneously with’ would be better than ‘parallel’. 

**Response: We removed all occurrences of J-scaling and replaced them with a more suitable term. We replaced parallel with simultaneously.**

 Reviewer: Figure 4: The cross-sectional vertical lines would be better in dashed format. Positive and negative contour colour differences are not explained. It might be worthwhile to comment on the asymmetric structure of the cross peak in the 13C dimension. Caption: there is no reference to A,B,C,D panel labels. The description of the pulse sequence scaling evolution delays needs to be improved (‘the J-scaling’ is shorthand; ‘the J-only evolution’ period might be better). 

**Response:** **This figure has been redesigned, addressing all points above.**

 Reviewer: Figure 5: A and B labels for the different figure elements are missing. Why are some of the 13C pulses shown in grey? I would prefer it if the two NMR spectra were not superposed, but rather plotted side by side. Caption: It is ambiguous whether the grey 15N pulse is employed along with the amber pulses. I am not keen on the use of colour in the exposition of the phase cycles. 

**Response:** **This figure (now figure 6) has been redesigned, removing colour from the spectrum plots.**

 Reviewer: Figure 6: Panel C seems to have more to do with A than B, so order is confusing. ‘n’, ‘n’’, ‘n’’’ ‘H[2]N’, ‘H[2]C[2]’ and ‘H[3]C[3]’ are not clearly explained (molecular structure needs numbering). The caption is in error when referencing B and C. Panel C switches from C[2] to C(2), etc. What do the different colour traces in panel C indicate? The caption does not explain. Note 1: on page 7 of the text there is reference to a panel D, and this strangle out of order as reference to panels A and B comes later. Note 2: Later the text describes the long range 1H 15N HSQC spectrum shown in panel C but without reference to it. It appears to describe the spectrum in terms of carbon C(2) and a two bond 2JNH coupling, but should properly be C(3) (Cbeta) and 2JNCbeta. Note 3: The text goes on (page 8) to discuss the metabolic pathway in a section that would more properly be in the Discussion, and then there is a short paragraph about Metabolab that should be in the Methods section. 

**Response:** **We addressed all these points.**

 Reviewer: When 1D spectra are mentioned (page 11) is this meant to refer to 13C 1D spectra?

**Response:** **This referred to 1D ^1^H spectra. This has been made explicit in the text now.**

 Reviewer: There is a claim for ‘unprecedented’ detail. The utility of J-scaling to reveal small couplings is not very well exemplified by the results, since no fitted cross sections are shown. Also whilst real-time measurements of metabolism using this approach can be anticipated, no (even synthetic) example is shown. The ‘High Speed’ moniker in the title for the manuscript is not strongly justified by the data shown, and not strongly emphasized by the account given. Would it not be preferable to provide side-by-side demonstrations of the effective information content, for a given complex sample* in (i) a 1D 1H-decoupled 13C spectrum; (ii) a non-aliased 2D 1H13C-HSQC spectrum with minimum phased cycle; (iii) an aliased version of (ii); and (iv) the ‘J-scaled’ versions of (ii) and (iii)? * of arbitrary complexity and concentration? 

**Response:** **We added spectra using accelerated acquisition. We acquired a 1D 13C spectrum, however we could not reach a sufficient signal to noise ratio given the amount of sample we had available. As we can reach acquisition times comparable to those for a 1D 1H spectrum and given that the analysis software would need substantial changes to be able to deal with aliased NMR spectra, we chose not to acquire aliased NMR spectra. The samples we acquired we believe are of sufficient complexity and variability (e.g. perfusion fluids as well as cell extracts).**

 Reviewer: Throughout the manuscript, greater care is required for the use of numerals instead of words (where appropriate, e.g. two scans, no 2 scans); spaces between numbers and units; avoidance of unexplained abbreviations (e.g. WIT-15); appropriate use of spectra/spectrum; avoidance of ambiguous orphan pronouns (e.g. ‘this/it’ [verb]); anthropomorphization (e.g. ‘cells form...’ page 10); it is D2O, not D20; almost invariably ‘spectrum’ should follow HSQC. I would be happy to proof read any revised version of the manuscript to help iron out any similar issues remaining. 

**Response:** **We checked all these carefully and changed the text accordingly.**

 Reviewer: In summary, whilst I see great value in the ideas presented here, and hope to employ them in our own future work, I believe that the authors could substantially upgrade the description of the new approach in a revised version of the manuscript.

# Peer review: Referee response for version 1

**DOI:** 10.21956/wellcomeopenres.14535.r29772

**Published:** 2018-01-23

**Authors:** Sebastien Serres

**Affiliations:** 1 University of Nottingham, Nottingham, UK

I did answer partly for 2 questions which do not alter the quality of this paper. My comments are listed below:

**General comments:**

 In this manuscript, Smith *et al.* present two techniques - quantitative spectral filters and enhanced J-coupling- that allow rapid collection of heteronuclear NMR data using HSQC NMR spectroscopy. The main advantage of these methods is that 2D- HSQC NMR spectra contain information about 1H-13C, 13C-13C and 13-15N coupling in a fraction of time it takes 1D spectra to acquire the same sample. Combining this NMR approach with metabolic modelling could be very powerful for quantify metabolic fluxes in cells or tissues.

 This paper is interesting and novel in terms of pulse-sequence but suffers lack of consistency and rationale regarding experimental approaches (i.e. biological models). Although the NMR approach using spectral filtering and enhanced J-coupling is clever, I don’t really understand why 3 biological models have been used in this paper. I understand that this work is a proof-of-concept but for consistency only the porcine kidney perfusion model should be used. In addition, this model is relevant to the primary aim of this study- the real-time tracing of metabolic pathways in whole organ prior to transplantation. Additional data with porcine kidney perfusion are needed to improve this paper or at least a good rationale for why authors have used tumour cells for 15N tracing. I believe that 13C-15N glutamine metabolism can be detected in their porcine kidney model. This will add strength and rigour to the paper.

 What is the variability in quantitative spectral filters and enhanced J-coupling between experimental repeats? The authors don’t state the number of repetitions per experiment, so the reproducibility cannot be assessed. I believe that the percentage incorporation of ^13^C and ^15^N into metabolites could be assessed in this study and thus should be presented. Did authors run their samples using conventional 1D 13C NMR (Rodrigues et al 2013 Front Neuroenergetics) or proton-observed carbon-edited (POCE) strategy (Rothman et al. 1985 PNAS) for comparison?   

**Specific comments:**

1. To avoid any confusion, authors should mention that NMR analysis is done *ex vivo* or *in vitro* but provides information on metabolic pathways *in vivo*.
2. Not sure about what authors mean by high-resolution data. Is it related to sensitive measurements of metabolites (~microM) or a better signal splitting with ultra-high field NMR? Or is it in comparison with ^13^C data collection using mass spectrometry?
3. Authors claim that” such techniques permit high throughput metabolic pathway profiling, increasing access, affordability and sensitivity when using NMR as an investigative modality”

Are these sequences freely available and easy to implement on Bruker NMR spectrometers? However not every sites in the world have access to cryoprobe.

1. “The standard unlabelled glucose constituent (10mM) within classical UW MPS was replaced with universally labelled glucose, at the same concentration, for use as a metabolic tracer”. How long was the labelling time for? 
2. To correct: “needed in in order to resolve J-couplings “
3. Please add a table that summarizes the pros and cons of their new techniques (e.g. acquisition time, SNR, J coupling enhancement etc…). This need to be compared with conventional 1D NMR spectra or POCE.
4. Figure 4 legend did not mention that the spectrum displays C(6) of 13C enriched glucose.
5. Why using a different NMR spectrometer for glutamine experiment (900 vs 600MHz)?

 “2D 1 H13C-HSQC and 2D 1 H15N-HSQC NMR spectra were acquired using a Bruker Avance III 900 MHz NMR spectrometer equipped with a 5mm z-PFG TCI Cryoprobe”

I have read this submission. I believe that I have an appropriate level of expertise to confirm that it is of an acceptable scientific standard, however I have significant reservations, as outlined above.

# Response

**Published:** 2018-08-17

**Authors:** Christian Ludwig

**Affiliations:** University of Birmingham, UK

We thank Sebastien Serres for his comments and suggestions. These have been very helpful to improve the quality of the manuscript.

 Reviewer: I did answer partly for 2 questions which do not alter the quality of this paper. My comments are listed below: 

 General comments: 

 In this manuscript, Smith et al. present two techniques - quantitative spectral filters and enhanced J-coupling- that allow rapid collection of heteronuclear NMR data using HSQC NMR spectroscopy. The main advantage of these methods is that 2D- HSQC NMR spectra contain information about 1H-13C, 13C-13C and 13-15N coupling in a fraction of time it takes 1D spectra to acquire the same sample. Combining this NMR approach with metabolic modelling could be very powerful for quantify metabolic fluxes in cells or tissues.

 This paper is interesting and novel in terms of pulse-sequence but suffers lack of consistency and rationale regarding experimental approaches (i.e. biological models). Although the NMR approach using spectral filtering and enhanced J-coupling is clever, I don’t really understand why 3 biological models have been used in this paper. I understand that this work is a proof-of-concept but for consistency only the porcine kidney perfusion model should be used. In addition, this model is relevant to the primary aim of this study- the real-time tracing of metabolic pathways in whole organ prior to transplantation. Additional data with porcine kidney perfusion are needed to improve this paper or at least a good rationale for why authors have used tumour cells for 15N tracing. I believe that 13C-15N glutamine metabolism can be detected in their porcine kidney model. This will add strength and rigour to the paper.

**Response: We acquired new data from a renal epithelial cell line. Unfortunately, whole organ perfusion experiments with ^13^C, ^15^N glutamine would be prohibitively expensive due to the amount of glutamine needed in the perfusion fluid. In addition, glutamine is not part of the formulation of KPS-1, so that the results obtained with such a modified perfusion solution would not lead to data directly comparable with clinical data.**

**We have simplified the biological side by concentrating more on kidney metabolism and removing the cancer cell line.**

 Reviewer: What is the variability in quantitative spectral filters and enhanced J-coupling between experimental repeats? The authors don’t state the number of repetitions per experiment, so the reproducibility cannot be assessed. I believe that the percentage incorporation of 13C and 15N into metabolites could be assessed in this study and thus should be presented. Did authors run their samples using conventional 1D 13C NMR (Rodrigues et al 2013 Front Neuroenergetics) or proton-observed carbon-edited (POCE) strategy (Rothman et al. 1985 PNAS) for comparison? 

**Response: As this is a proof of concept paper, we did not run biological replicates. We expect that the reproducibility will be similar to our recently published paper in Angewandte Chemie, where we demonstrate benefits of combining NMR and MS data. The error margins obtained there were very small, resulting in correlation coefficients very close to 100% when comparing experimental and simulated signals.**

**We acquired a 1D ^13^C NMR spectrum from one of the samples. In order to obtain the best signal to noise ratio, we diluted the sample from 30 ul to 600 ul and acquired the carbon spectrum using a 5 mm TXO cryoprobe, which is optimised for ^13^C detection. As expected the signal to noise ratio was much poorer in the 1D 13C spectrum compared to the HSQC spectrum, despite a very long acquisition time for the ^13^C 1D spectrum. We added the ^13^C 1D spectrum to the deposited data.**

**We also acquired POCE spectra, which require the subtraction of two very large signals to obtain the amount of ^13^C incorporation. This can result in significant subtraction artefacts, which are not seen in our approach because of the use of pulsed field gradients. We included a brief discussion in the main text and added the spectra to the deposited data.**

 Reviewer: Specific comments: 

1. Reviewer: To avoid any confusion, authors should mention that NMR analysis is done ex vivo or in vitro but provides information on metabolic pathways in vivo. 

 

**Response: We added a statement to this effect to the main text.**

  

1. Reviewer: Not sure about what authors mean by high-resolution data. Is it related to sensitive measurements of metabolites (~microM) or a better signal splitting with ultra-high field NMR? Or is it in comparison with 13C data collection using mass spectrometry? 

 

**Response: We were referring to the ability to resolve metabolic pathway utilisation. We added a statement to this effect to the abstract.**

  

1. Reviewer: Authors claim that” such techniques permit high throughput metabolic pathway profiling, increasing access, affordability and sensitivity when using NMR as an investigative modality” 

 

**Response: We added a new figure that demonstrates the acceleration of acquisition.**

 Reviewer: Are these sequences freely available and easy to implement on Bruker NMR spectrometers?

**Response: In accordance with the Wellcome openresearch journal’s rules, all sequences are freely available. They have been designed to comply with Bruker’s pulse sequence nomenclature so that a standard setup is easily possible.**

 Reviewer: However not every sites in the world have access to cryoprobe. 

**Response: It may be possible to run all pulse sequences on room temperature probes. However, as we have not tested the sequences on RT probes, we can’t give recommendations. We included a short discussion mentioning RT probes in the text.**

  

1. Reviewer: “The standard unlabelled glucose constituent (10mM) within classical UW MPS was replaced with universally labelled glucose, at the same concentration, for use as a metabolic tracer”. How long was the labelling time for? 

 

**Response: We added this information**

  

1. Reviewer: To correct: “needed in in order to resolve J-couplings “ 

 

**Response: Corrected**

  

1. Reviewer: Please add a table that summarizes the pros and cons of their new techniques (e.g. acquisition time, SNR, J coupling enhancement etc...). This need to be compared with conventional 1D NMR spectra or POCE.

 

**Response: We added such a table to the main text.**

  

1. Reviewer: Figure 4 legend did not mention that the spectrum displays C(6) of 13C enriched glucose.

 

**Response: This has been corrected.**

  

1. Reviewer: Why using a different NMR spectrometer for glutamine experiment (900 vs 600MHz)? “2D 1 H13C-HSQC and 2D 1 H15N-HSQC NMR spectra were acquired using a Bruker Avance III 900 MHz NMR spectrometer equipped with a 5mm z-PFG TCI Cryoprobe”

 

**Response: We simplified the biology of the samples and now acquired all spectra used for this paper at 600 MHz.**
